# World economies’ progress in decoupling from CO_2_ emissions

**DOI:** 10.1038/s41598-024-71101-2

**Published:** 2024-09-03

**Authors:** Jaume Freire-González, Emilio Padilla Rosa, Josep Ll. Raymond

**Affiliations:** 1 Institute for Economic Analysis (CSIC) and Barcelona School of Economics, 08193 Bellaterra, Barcelona, Spain; 2 Department of Applied Economics, Univ. Autonoma de Barcelona, 08193 Bellaterra, Spain; 3 Department of Economics and Economic History, Univ. Autonoma de Barcelona, 08193 Bellaterra, Spain

**Keywords:** Decoupling, CO_2_ emissions, Economic growth, Environmental Kuznets curve, Multicollinearity, Segmented-sample regressions, Climate-change mitigation, Climate-change policy, Environmental economics, Environmental impact, Sustainability

## Abstract

The relationship between economic growth and CO_2_ emissions has been analyzed testing the environmental Kuznets curve hypothesis, but traditional econometric methods may be flawed. An alternative method is proposed using segmented-sample regressions and implemented in 164 countries (98.34% of world population) over different periods from 1822 to 2018. Results suggest that while the association between GDP per capita and CO_2_ emissions per capita is weakening over time, it remains positive globally, with only some high-income countries showing a reversed association in recent years. While 49 countries have decoupled emissions from economic growth, 115 have not. Most African, American, and Asian countries have not decoupled, whereas most European and Oceanians have. These findings highlight the urgency for effective climate policies because decoupling remains unachieved on a global scale, and we are moving away from, rather than approaching, the Paris Agreement goal of limiting temperature increase to 1.5 °C above preindustrial levels.

## Introduction

The relationship between economic growth and environmental impacts has been extensively studied in the economic literature. In the last decades the debate has mostly focused on the environmental Kuznets curve (EKC)^1,2^, an optimistic hypothesis on the relationship between environmental impacts and gross domestic product (GDP) per capita. The EKC posits that in the early stages of development, economic growth leads to an increase in environmental degradation, but after achieving a certain GDP per capita threshold or turning point, environmental degradation decreases with economic growth^3^. That is, it hypothesizes an inverted-U or bell-shaped relationship between GDP per capita and environmental degradation. The EKC hypothesis was initially proposed by Grossman and Krueger^3^, while Panayotou^4^ was the first to coin the term EKC due to its similarity to the relationship that Kuznets^5^ suggested between income inequality and per capita income. Some empirical studies have supported the hypothesis for specific pollutants, resources use, or impacts in some countries or groups of countries, but there is also much evidence rejecting it for several environmental degradation indicators, countries, and time frames, so the EKC cannot be considered a general pattern^6^. Empirical studies have also identified other patterns, like linear relationships^7^, U-shaped relationships^8^, or patterns that include two turning points starting to rise or decrease again after a specific GDP per capita level. These can adopt an N-shaped form^9–11^ or an inverted N-shaped form^12^. Most of the studies on the EKC hypothesis are based on atmospheric indicators, whereas there is more limited evidence on other pollutants or resources, like land oceans, seas, coasts and biodiversity indicators, and freshwater indicators^13^.

In relation to climate change, there is some controversy on the form of the relationship between GDP per capita and greenhouse gas emissions. Studies have used different methodologies to empirically test the shape of the relationship, particularly for carbon dioxide (CO_2_) emissions. Some studies focus on single country analysis^7,8,14–18^, while some of them direct their analyses on groups of countries^11,19–28^, with a diversity of methods and results. Some recent literature reviews summarize well this literature. Shahbaz and Sinha^29^ found that the literature on the EKC for CO_2_ emissions is inconclusive, which may be caused by the different contexts, time periods, explanatory variables, and methods employed. They suggest that studies should focus on new explanatory variables and refining the data set. Sarkodie and Strezov^13^, using meta-analysis methods, found heterogeneity among the GDP per capita level of the turning point in studies that validate the EKC hypothesis due to differences in the period of study and econometric methods used in model estimation.

Our paper aims to determine whether there is a decoupling between GDP and CO_2_ emissions, while avoiding the methodological issues of previous studies. This decoupling is the focus of the EKC literature. Although there are other approaches like convergence or inequality analysis^30–33^ that also evaluate the evolution between GDP and CO_2_, they address different research questions than ours and the EKC literature, which specifically examines this decoupling.

Most studies that empirically test the EKC hypothesis suffer from shortcomings that can affect the conclusions drawn from literature reviews. As pointed out by Jardon et al.^21^, the empirical validity of EKC studies has been questioned due to the sensitivity of the results to variations in model specifications, lack of diagnosis of the stationarity properties of the variables, assumption of cross-sectional independence (for panel data), and possible presence of structural breaks in the long-run relationship implied by the EKC hypothesis (for details see Refs.^34–37^). Furthermore, numerous studies are constrained by limited time frames, potentially capturing only short-term or time-specific effects, or are limited to specific geographic areas, failing to account for transboundary effects, for instance. Not all studies conduct all needed statistical verifications to provide robust results. Another critical limitation of the EKC literature is that studies analyzing panels or cross-section data of countries impose the same functional form and parameters for the entire set of countries in estimations. This assumption has been rejected in some studies when tested^25–38^. In fact, the relation estimated in the literature for panels of countries may be the result of the juxtaposition of different trajectories of various countries^25^. Therefore, even if some studies find evidence supporting the EKC for a panel of countries, it may not provide valid conclusions on the expected path for the different countries. Individual analysis is essential to understand the dynamics and characteristics of each country^7,43^.

Another important shortcoming, not usually acknowledged in literature, is the potential existence of multicollinearity in estimated regressions^44^. It tends to appear when a regressor is specified along with its squared and/or cube form in a multiple regression model. It does not necessarily reduce the predictive power of the model as a whole but affects the estimations of individual parameters. As individual values assigned to different (GDP per capita) parameters are key in determining the functional form of the relationship between GDP per capita and pollution, multicollinearity may lead to estimate wrong curves. Some authors have considered it and provided alternative estimation methods as a solution. In this sense, Narayan and Narayan^24^ compare short- and long-run elasticities of GDP on CO_2_ emissions for some developing countries. They assume that if short-run estimates are larger than long-run ones, more income will lead to fewer CO_2_ emissions, and the other way round. Al-Mulali et al.^15^ also used this approach to avoid multicollinearity for estimates for Kenya. However, this method does not allow to track the functional form for a whole period, and it is not well suited to examine decoupling, which does not depend on the differences between long- and short-term elasticities but on the existence of a negative long-term elasticity.

This study aims to examinate the relationship between CO_2_ emissions and economic growth across a comprehensive set of 164 countries over different time periods, with a focus on the 1822–2018 period. The countries included in the sample accounted for 98.34% of the total world population in 2018, representing 7534 million people, with only a few small countries discarded from the sample due to data limitations. The study covers the most extensive time frame in studies of this nature to date. Most important, we address the limitations of previous studies and avoid the methodological flaws that may have led to inferring incorrect patterns. The motivation of the study is to contribute to the EKC debate by: (1) correcting errors that previous studies may have committed by not considering the serious problems multicollinearity cause in estimated parameters; (2) proposing a new econometric approach, which avoids the mentioned problem from previous methods in estimating the relationship between CO_2_ emissions and economic growth; (3) providing estimates for most of the countries of the world with this method, in a homogenous framework.

First, we apply an exhaustive statistical and verification analysis to provide robust results, which includes diagnosis of multicollinearity and diagnosis of the stationarity and cointegration properties of variables. Second, we apply segmented-sample regressions for individual countries, an alternative estimation method consisting in splitting the samples in smaller periods for each country and regressing GDP per capita on CO_2_ per capita. A total of 932 regressions have been conducted. This way we are able to find elasticities for each country and period, and therefore, we build sequentially the curve for each country without imposing a priori assumptions on the functional form, such as including a quadratic form of GDP per capita and allowing the data to speak by themselves. The large data set used, along with the proposed methodological approach, allows us to provide robust evidence and overcome the shortcomings incurred in previous studies.

We find that the relationship between CO_2_ emissions per capita and GDP per capita is still positive on average. However, there is a global tendency toward the weakening of this relationship. Therefore, decoupling of economic growth and CO_2_ has not yet been achieved on a global scale. The article is structured as follows: section "Methods" describes the flaws of the methods used in literature, as well as the methods used in this research; section "Results" shows the results obtained in different ways, considering global and individual results. Section "Discussion" discusses the main results in relation to the state-of-art of the topic, and section "Conclusion" contains the main conclusions of this research.

## Methods

The first part of the methodology consists in performing multicollinearity analysis between regressors as well as unit root and cointegration tests to the series to avoid spurious regressions and test the existence of a long-run relationship between the relevant variables for the analysis. The second part of the methodology consists in performing multiple individual regressions, for each country in different periods (segmented-sample regressions), to test the sign and intensity of the relationship between CO_2_ emissions and economic growth. The tested specification is a simple model relating CO_2_ emissions per capita with GDP per capita: $CO{2}_{cap}=f(GD{P}_{cap})$. Unlike some previous studies, we have not included other control variables often found in literature like urban population, trade openness, and energy use^45^. The exclusion of the former two is due to a potential problem of overparameterization of the model because we have a limited number of observations, as we will detail later; the latter, in addition to the issue of overparameterization, is also for the consideration that CO_2_ and energy use are coupled variables^21^, as for the years covered in this analysis, most of the primary energy comes from fossil sources. This happens when a variable directly or indirectly contains the whole or components of another variable, leading to invalid conclusions if they are included in the same regression equation (Archie Jr.^46^). In addition, the use of a reduced form model captures all the direct and indirect relationship between CO_2_ and GDP per capita, including effects associated to omitted variables that may be correlated with both economic activity and time^47^, so including additional variables could potentially distort the analysis^38^. Although this is the appropriate analysis to study apparent elasticities between the variables, it does not allow us to assess the causes of the relationship or the determinants of emissions^25^.

### Data

We have obtained a data set for 164 countries for the period 1822–2018 for three variables: GDP, production-based CO_2_ emissions, and population. However, data are not available for all the years of the period in all countries. GDP is measured in international dollars using 2011 prices to adjust for inflation and price differences between countries. It has been obtained from the Maddison Project Database^48^. Production-based CO_2_ emissions are measured in million tons and include all emissions from energy production (coal, oil, natural gas and flaring) as well as industrial emissions from cement and steel production. They do not include emissions from land use change. This variable is obtained from the Global Carbon Project^49^. Population is used to calculate per capita GDP and CO_2_ emissions. From 1800 to 1949 data on population comes from historical estimates by Gapminder v7. From 1950 to 2018 population records are by the United Nations Population Division (2018). Appendix I contains the main descriptive statistics of the two main variables included in the regressions for all the countries: production-based CO_2_ emissions per capita and GDP per capita.

### Multicollinearity

#### Unreliable individual EKC estimators with multicollinearity

Classical model specifications of the EKC are as follows:

1 $$\text{ln}{\left(CO{2}_{cap}\right)}_{it}={\alpha }_{it}+{\beta }_{1}\text{ln}{(GD{P}_{cap})}_{it}+{\beta }_{2}\text{ln}{(GD{P}_{cap})}_{it}^{2}+{\beta }_{3}\text{ln}{(GD{P}_{cap})}_{it}^{3}+{\varepsilon }_{it}$$

where $CO{2}_{cap}$ is CO_2_ emissions per capita, and $\mathit{GD}{P}_{\mathit{cap}}$ is GDP per capita. Additionally, specifications include ${(GD{P}_{cap})}_{it}^{2}$ to capture a quadratic relationship and the turning point in the curve and, in some cases, also ${(GD{P}_{cap})}_{it}^{3}$ to capture more complex polynomial functions. The signs of parameters determine the functional form of the relationship^10,12^: if ${\beta }_{1}={\beta }_{2}={\beta }_{3}=0$, there is either a flat pattern or no relationship between CO_2_ and GDP; if ${\beta }_{1}>0$ and ${\beta }_{2}={\beta }_{3}=0$, there is a monotonic increasing relationship between both variables; if ${\beta }_{1}<0$ and ${\beta }_{2}={\beta }_{3}=0$, there is a monotonic decreasing relationship; if ${\beta }_{1}>0$ and ${\beta }_{2}<0$ and ${\beta }_{3}=0$ there is an inverted U-shaped relationship, that is, an EKC; if ${\beta }_{1}<0$ and ${\beta }_{2}>0$ and ${\beta }_{3}=0$, there is a U-shaped curve; if ${\beta }_{1}>0$ and ${\beta }_{2}<0$ and ${\beta }_{3}>0$, there is an N-shaped relationship; if ${\beta }_{1}<0$ and ${\beta }_{2}>0$ and ${\beta }_{3}<0$, there is an inverted N-shaped relationship.

Multicollinearity occurs when there is a high correlation between explanatory variables. The extreme multicollinearity that this formulation introduces can result in unreliable parameter estimates, invalidating the conclusions on individual estimators. To test multicollinearity, we use the variance inflation factor (VIF) as a measure of its degree. In our sample of countries, using a quadratic form of the GDP per capita as explanatory variables, frequent VIF values exceeding 800 were observed. These results suggest the presence of extreme multicollinearity, indicating that the estimated individual coefficients are unreliable in all cases.

Because multicollinearity affects estimated values of individual coefficients, we have developed an alternative approach instead of testing the specifications by including one turning point fitting a quadratic form (U-shaped or inverted U-shaped curves) or two turning points fitting a cubic form (testing the N-shaped or the inverted N-shaped form).

#### Problems with previous methods in the literature to avoid multicollinearity in estimations testing the EKC hypothesis

Some previous studies have proposed alternative methods for testing the EKC hypothesis to avoid the multicollinearity problem. Particularly, Narayan and Narayan^24^ proposed an alternative way of judging if countries had reduced CO_2_ emissions over time with growth in incomes by comparing the short- with the long-run income elasticities. They argue that if the latter is smaller than the former, GDP per capita growth will eventually lead to less CO_2_ emissions. However, this is not necessarily correct because emissions per capita will increase in the long run if the long-run elasticity between emissions and GDP per capita is greater than zero and will only decrease if this long-run elasticity is lower than zero. This method is neither suited to test a weak decoupling—a decrease in the emissions per unit of GDP—because it needs a long-run elasticity lower than one independently on the difference between short- and long-run elasticities.

Consider a general dynamic model of the type:

2 $$\alpha \left(L\right)\mathit{ln}{{CO2}_{cap}}_{t}\text{ } = \mu + \text{ } \beta \left(L\right)\mathit{ln}{{Y}_{cap}}_{t}+{u}_{t}$$

where $\alpha \left(L\right)$ and $\beta \left(L\right)$ are polynomials in the lag operator *L*, $\mathit{ln}{{CO2}_{cap}}_{t}$ is the logarithm of CO_2_ emissions per capita, and $\mathit{ln}{{Y}_{cap}}_{t}$ is the logarithm of GDP per capita. Given that the model can be expressed as follows:

3 $$ln{CO2}_{cap,t}=\mu +{\alpha }_{1}ln{CO2}_{cap,t-1}+\cdot \cdot \cdot \cdot \cdot +{\alpha }_{r}ln{CO2}_{cap,t-r}+{\beta }_{0}\mathit{ln}{Y}_{cap,t}+\cdot \cdot \cdot \cdot +{\beta }_{s}\mathit{ln}{Y}_{cap,t-s}+{u}_{t}$$

Short-term elasticity is ${\beta }_{0}$, whereas $\frac{{\beta }_{0}+{\beta }_{1}\cdot \cdot \cdot +{\beta }_{s}}{1-{\alpha }_{1}\cdot \cdot \cdot -{\alpha }_{r}}=k$ is the long-term emissions–GDP per capita elasticity.

If an economy grows at a stable rate of $\dot{Y}_{cap}$, CO_2_ emissions per capita will also grow at a stable rate of $k\dot{Y}_{cap}$. If *k* > 1, emissions per unit of GDP will tend to increase regardless of whether ${\beta }_{0}>k$ or ${\beta }_{0}<k$ is verified.

Therefore, the key factor determining the increase or decrease in CO_2_ emissions per unit of GDP is whether the long-run elasticity is greater or smaller than 1. Moreover, CO_2_ emissions per capita only decrease with an increase in GDP per capita if the long-run elasticity is negative. This is the requisite for decoupling, which does not depend on the difference between short- and long-run elasticities but on the sign of long-run elasticity.

### Stationarity and cointegration

To ensure that regression models are not spurious, it is necessary to conduct a unit root test analysis of the random disturbance term, testing its stationarity. If the variables are not stationary but are cointegrated, it can be concluded that they have a long-term relationship. In this section, we first conduct panel unit root tests for all the countries of the analysis for CO_2_ per capita and GDP per capita. Then, we also conduct different cointegration tests.

We specifically use six panel unit root tests to evaluate the stationarity of the series: the Levin–Lin–Chu (LLC) test^50^, Breitung t-statistic^51^, Hadri Z-statistic^52^, Im–Pesaran–Shin W-statistic (IPS)^53^, Fisher-augmented Dickey–Fuller (ADF–Fisher); (Maddala and Wu^54^), and Fisher– Phillips and Perron (PP-Fisher)^55^. The LLC, Hadri, and Breitung assume a common unit root process, whereas the IPS, ADF–Fisher and PP–Fisher assume an individual root process. Differences between tests in the context of the EKC literature are detailed in Jardón et al^21^. We have conducted all tests in two ways: with constant (intercept) and with constant plus trend, except Breitung, which is only allowed with trend and intercept. Results of the tests are shown in Table 1.

**Table 1:** Panel unit root tests.

		CO_2_ per capita	$\Delta$ CO_2_ per capita	GDP per capita	$\Delta$ GDP per capita
Levin–Lin–Chu test	Intercept	7.93178 (1.0000)	− 145.893 (0.0000)	65.7347 (1.0000)	− 64.6426 (0.0000)
	Trend and intercept	6.02640 (1.0000)	− 161.448 (0.0000)	31.5961 (1.0000)	− 81.4037 (0.0000)
Hadri Z-stat	Intercept	70.9857 (0.0000)	− 7.99289 (1.0000)	74.7347 (0.0000)	18.9560 (0.0000)
	Trend and intercept	60.5596 (0.0000)	− 10.5244 (1.0000)	72.2966 (0.0000)	12.3811 (0.0000)
Breitung t-stat	Trend and intercept	14.9633 (1.0000)	− 75.6435 (0.0000)	47.0664 (1.0000)	− 49.4625 (0.0000)
Im–Pesaran–Shin W-stat	Intercept	8.37339 (1.0000)	− 141.121 (0.0000)	61.0908 (1.0000)	− 66.8121 (0.0000)
	Trend and intercept	4.15549 (1.0000)	− 145.185 (0.0000)	36.6776 (1.0000)	− 73.5614 (0.0000)
ADF–Fisher Chi-square	Intercept	448.868 (0.3747)	9283.45 (0.0000)	65.3942 (1.0000)	4554.95 (0.0000)
	Trend and intercept	542.842 (0.0006)	9751.08 (0.0000)	74.9949 (1.0000)	4569.31 (0.0000)
PP–Fisher Chi-square	Intercept	436.283 (0.5411)	9392.56 (0.0000)	64.9452 (1.0000)	4730.41 (0.0000)
	Trend and intercept	553.258 (0.0002)	10,294.7 (0.0000)	93.5527 (1.0000)	4617.18 (0.0000)

For all tests except for Hadri, the null hypothesis is non-stationarity. Lag lengths are selected automatically with the Akaike information criterion (AIC); p-values in parentheses.

Source: own elaboration.

Based on the different tests we conducted, we can conclude that, in general, CO_2_ per capita and GDP per capita are non-stationary at levels, having a unit root. Therefore, we can consider them as integrated processes of order 1.

We investigate the existence of a long-term relationship between the two variables through cointegration tests. We employ three different panel cointegration tests: Pedroni^56,57^, Kao^58^, and a Fisher-type test using an underlying Johansen methodology^54^. Following Pedroni^56^, we estimate the different statistics of his test including a constant and a trend because they are more reliable than just including a constant. The Kao test follows the same approach as the Pedroni but is based on the assumption of homogeneity across the panel. Kao^58^ derives two (DF and ADF) types of panel cointegration tests. The results of the cointegration tests are presented in Table 2.

**Table 2:** Cointegration tests.

	Statistic	Weighted statistic
**Pedroni residual cointegration test**		
Alternative hypothesis: common AR coefficients (within-dimension)		
Panel v-Statistic	1.533926 (0.0625)	− 6.533586 (1.0000)
Panel rho-Statistic	− 74.19208 (0.0000)	− 3.595931 (0.0002)
Panel PP-Statistic	− 66.40794 (0.0000)	− 0.958834 (0.1688)
Panel ADF-Statistic	− 68.73749 (0.0000)	2.416838 (0.9922)
Alternative hypothesis: individual AR coefficients (between-dimension)		
Group rho-Statistic	− 2.789044 (0.0026)	
Group PP-Statistic	− 2.603061 (0.0046)	
Group ADF-Statistic	− 3.144187 (0.0008)	
**Kao Residual Cointegration Test**		
ADF	2.586627 (0.0048)	

	Fisher Statistic* (from trace test)	Fisher Statistic* (from max-eigen test)
**Johansen Fisher Panel Cointegration Test**		
Trend assumption: No deterministic trend		
None	1708 (0.0000)	1639 (0.0000)
At most 1	432.0 (0.0001)	432.0 (0.0001)
Trend assumption: No deterministic trend (restricted constant)		
None	1539 (0.0000)	1525 (0.0000)
At most 1	351.4 (0.1796)	351.4 (0.1796)
Trend assumption: Linear deterministic trend		
None	1224 (0.0000)	1183 (0.0000)
At most 1	493.1 (0.0000)	493.1 (0.0000)
Trend assumption: Linear deterministic trend (restricted)		
None	1383 (0.0000)	1070 (0.0000)
At most 1	245.0 (0.9998)	245.0 (0.9998)
Trend assumption: Quadratic deterministic trend		
None	829.9 (0.0000)	734.5 (0.0000)
At most 1	631.2 (0.0000)	631.2 (0.0000)

For Pedroni and Kao tests the null hypothesis is no cointegration. Results of Pedroni tests are estimated with deterministic intercept and trend.

*Probabilities are computed using asymptotic Chi-square distribution.

Source: own elaboration.

Results of cointegration tests in Table 2 show that mostly both variables are cointegrated, so they both have a long-term relationship.

### Segmented-sample regressions of the relationship between CO_2_ emissions per capita and GDP per capita

The application of unit root panel data tests of integration and cointegration is suitable if the relationships that links the different variables are relatively similar among countries. But if this is not the case, a simple technique, the segmented-sample regression approach, is more flexible to encompass the individual specificities of the countries included in the sample, which is our case. Applying rolling regressions^59^ would lead to similar conclusions but will require a much larger number of regressions. So, the third step of the methodology consists in performing this method to estimate the relationship between CO_2_ emissions per capita and GDP per capita. For each country, we estimate successive regressions for periods of 15 years (*T*), starting at 2018 and going backward, taking the first year of the previous regression as the last year of the current one. We choose this time frame to have at least two regressions (periods) for all countries and be able to compare them because not all series of countries have the same total length. Harrell^60^ assumes that it is necessary to have at least 10–20 observations per parameter estimated to be able to detect effects with a reasonable statistical power. Periods with fewer than 10 observations have not been regressed. Therefore, for each period *T*, we choose this simple functional form with just one regressor. This avoids both multicollinearity existent in polynomial functional forms, and the issue of overparameterization in regression models with few observations:

4 $$\text{ln}{\left(CO{2}_{cap}\right)}_{it}={\alpha }_{it}+{\beta }_{it}\text{ln}{({Y}_{cap})}_{it}+{\varepsilon }_{it}$$

where ${\beta }_{1}$ represents the income elasticity of CO_2_ of emissions for country *i* in year *t*. The objective for each country is to obtain different elasticities for different periods and observe their changes to determine the form of the relationship. We estimate them by ordinary least squares using the Newey–West estimator^61^, also known as HAC (heteroskedasticity and autocorrelation consistent), to estimate the standard errors of the coefficients. We have performed a total of 932 regressions for different periods for the 164 countries. Table 3 summarizes the regressions carried out.

**Table 3:** Summary of regressions.

Number of periods of 15 years regressed (*T*)	Number of countries (*N*)	Number of regressions (T*N)
2	5	10
3	16	48
4	20	80
5	83	415
6	4	24
7	7	49
8	5	40
9	6	54
10	4	40
11	4	44
12	5	60
13	2	26
14	3	42
TOTAL	164	932

Source: own elaboration.

Different possibilities can arise with the different results of the income-elasticities: if ${\beta }_{iT}>0$, there is a positive relationship between CO_2_ per capita and GDP per capita in period *T*, whereas if ${\beta }_{iT}<0$, there is a negative relationship between both variables. The inverted U-shaped relationship (EKC) will appear if ${\beta }_{iT}>0$ and ${\beta }_{iT+1}<0$; an N-shaped relationship will happen if ${\beta }_{iT}>0$, ${\beta }_{iT+1}<0$, and ${\beta }_{iT+2}>0$. If ${\beta }_{iT}>{\beta }_{iT+1}>0$, there is a growing relationship between both variables along the whole period, although they grow less in successive periods.

## Results

The results of the regressions can be summarized in several ways, given the vast number of elasticities obtained for different countries and time periods. The 932 elasticities are grouped to present global results and individual results. For detailed results in all countries and periods, see Appendix II, which contains a table with the results of estimates for each country and period, providing a total of 932 elasticities. Furthermore, Appendix III provides visual representations of the estimated elasticity curves for each one of the 164 countries.

### Global results

**Fig. 1:**
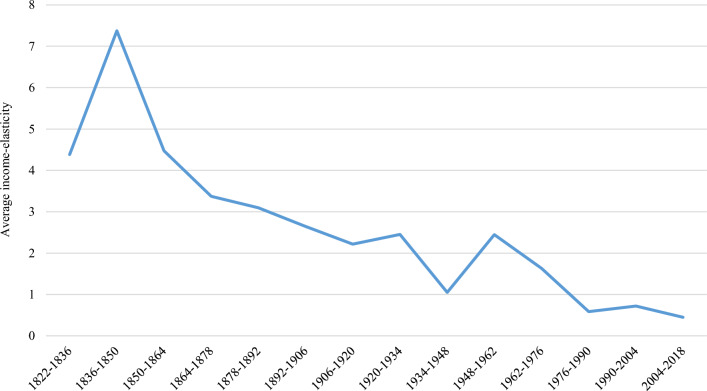
Average income elasticity of CO_2_ of the 164 countries analyzed in each period.

Figure 1 illustrates the temporal evolution of the average income elasticity between CO_2_ emissions per capita and GDP per capita over the entire period of analysis. The trend reveals a consistently positive relationship between the two variables, although it becomes weaker over time. Specifically, in the period 2004–2018 CO_2_ per capita grew along with GDP per capita but at a slower rate than in previous periods. Because the average elasticity in this last period is between zero and one, CO_2_ emissions per capita grow less than GDP per capita when the latter grows, but they still have a positive relationship. This is sometimes known in the literature as “weak” decoupling. Although the trend does not indicate an inverted U-shape in average, it is important to interpret this figure with caution because the sample sizes differ across time frames and because we plot average elasticities from very different economies.

**Fig. 2:**
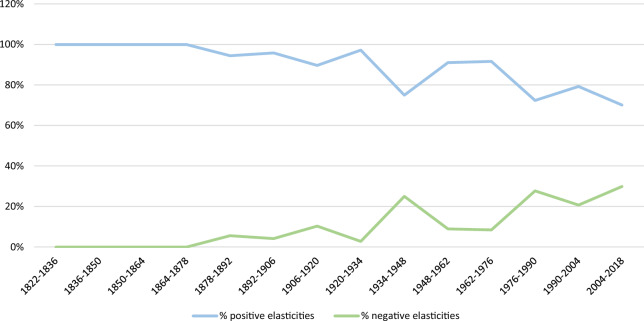
Percentage of the number of positive and negative elasticities in each period for the 164 countries.

The signs of elasticities play a pivotal role in determining the functional form of the relationships between CO_2_ and GDP per capita for each country. Positive elasticities mean a positive association between economic growth and emissions, reflected in the positive slopes of the curves, whereas negative elasticities indicate a negative relationship, evidenced by the negative slopes of curves. Focusing on the signs, Fig. 2 disaggregates the number of positive and negative elasticities in each period regardless of their value, showing a convergence between the two kinds of elasticities along the period. However, there are still more countries with positive elasticities in 2004–2018 than those with negative ones.

**Fig. 3:**
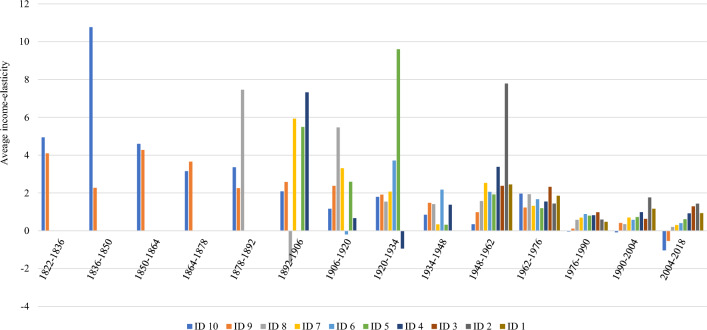
Average income elasticity of CO_2_ by period and GDP per capita decile. ID means income decile. ID 1 represents the lowest GDP per capita, whereas ID 10 is the highest.

We have also grouped countries according to their GDP per capita in 2018 into 10 deciles, so we have all countries ordered from higher to lower income per capita. Then we have calculated the average elasticity within each group during the periods analyzed. The results of the analysis are presented in Fig. 3. Notably, only the richest countries achieve negative elasticities on average in the most recent period of analysis (2004–2018) and partially in the previous period. However, the general trend is toward reducing average elasticities. Some peculiar patterns appear before the period 1962–1976. This is attributable to a reduced number of observations available as we go further back through the different periods. Furthermore, potential biases may arise due to less reliable data for older periods and developing countries. Additionally, because we use 2018 GDP per capita, some countries would be assigned to different deciles in relation to 2018 classification as we go backward in time. We observe, for instance, that there is a negative elasticity in decile 4 for the period 1920–1934. This is because it is based on only two observations in that period (Philippines and India), so the results obtained for those countries carried more weight than observations for other countries in other deciles/periods.

**Fig. 4:**
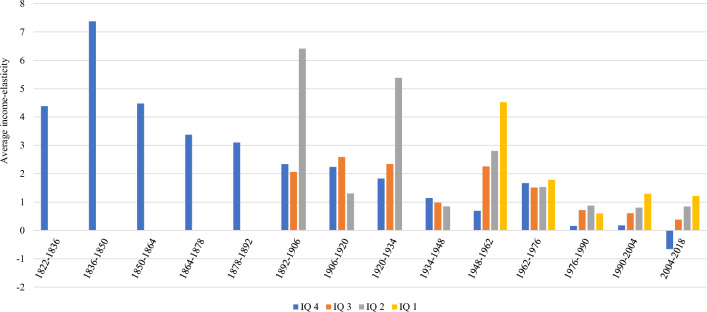
Average income elasticity of CO_2_ by period and GDP per capita quartile. IQ means income quartile. IQ 1 represents the lowest GDP per capita, whereas IQ 4 is the highest.

When we classify them by quartiles of GDP per capita, some noise disappears, and we observe that the wealthiest countries, and only in the last period (2004–2018), have a negative average elasticity. However, we observe the same tendency as in the analysis by deciles toward the reduction of elasticities in all quartiles over the period (Fig. 4).

### Individual results

We have classified all countries into six different groups based on the shape of the estimated relationship (see Table 4). The first three groups reveal “decoupling shapes,” where the last period or periods of analysis have an inverse relationship between CO_2_ per capita and GDP per capita. These shapes are a degrowing, an inverted U-shape (EKC curve) and an inverted N-shape. In contrast, we label the last three groups of countries as “non-decoupling shapes” because they show a positive relationship between the two variables in the last period(s) under analysis. Among the countries analyzed, 49 countries exhibit decoupling shapes, representing the 29.88% of the countries, whereas 115 have non-decoupling ones, which represents the 70.12%.

**Table 4:** Classification countries by the functional form of the relationship, sorted by 2018 GDP per capita.

**Decoupling shapes:**	
No of countries: 49 (29.88%)	
Population: 1490 million (19.79%)	
GDP: 47,700 billion dollars (42.11%)	
Average GDP per capita: $32,227	
Degrowing shaped:	Slovakia
Inverted U-shaped:	Norway, Switzerland, Luxembourg, United States, Hong Kong, Australia, Netherlands, Germany, Denmark, Sweden, France, Japan, United Kingdom, Israel, Malta, Czechia, Slovenia, Poland, Portugal, Hungary, Croatia, Romania, Bulgaria, Azerbaijan, Costa Rica, Serbia, North Macedonia, Uzbekistan, Jordan, El Salvador, Eswatini, Angola, Nigeria, Djibouti, North Korea, Togo, Malawi, Burundi
Inverted N-shaped:	Qatar, Singapore, Ireland(*), Canada(*), Iceland(*), Austria(*), Belgium(*), New Zealand(*), Mexico(*), South Africa(*)
**Non-decoupling shapes:**	
No of countries 115 (70.12%)	
Population: 6043 million (80.21%)	
GDP: 65,573 billion dollars (57.89%)	
Average GDP per capita: $12,936	
Growing shaped:	Taiwan, Italy, Spain, Seychelles, Russia, Malaysia, Turkmenistan, Trinidad and Tobago, Cyprus, Uruguay, Montenegro, Belarus, Iran, Lebanon, Thailand, Georgia, Dominican Republic, Equatorial Guinea, Botswana, China, Barbados, Bosnia and Herzegovina, Albania, Egypt, Sri Lanka, Armenia, Indonesia, Tunisia, Ecuador, Paraguay, Saint Lucia, Ukraine, Namibia, Dominica, Morocco, Cuba, Guatemala, Jamaica, Myanmar, Pakistan, Palestine, Honduras, Nicaragua, Tajikistan, Cote d'Ivoire, Cambodia, Syria, Nepal, Lesotho, Benin, Uganda, Comoros, Burkina Faso, Zimbabwe, Mali, Democratic Republic of Congo
U-shaped:	Lithuania, Kazakhstan, Latvia, Greece, Mauritius, Brazil, Moldova, Kyrgyzstan, Bangladesh, Tanzania, Senegal, Chad, Niger
N-shaped:	United Arab Emirates, Kuwait, Saudi Arabia, Bahrain, Finland, South Korea, Oman, Estonia, Chile, Panama, Turkey, Argentina, Gabon, Libya, Algeria, Colombia, Mongolia, Iraq, Peru, Venezuela, Philippines, Vietnam, Cape Verde(*), Laos, Bolivia(*), India, Congo, Ghana, Sao Tome and Principe, Kenya, Zambia, Mauritania, Cameroon, Yemen, Afghanistan, Rwanda, Ethiopia, Haiti(*), Gambia, Guinea, Guinea-Bissau, Madagascar, Sierra Leone, Mozambique, Liberia, Central African Republic

Population, GDP, and GDP per capita data are from 2018. Despite some countries having more than three changes of slope, we only take the last three to assign it a shape. The intensity and periods of the slopes vary between countries and are not reflected in this table.

(*) next to the country denotes more than 3 slope changes.

Source: own elaboration.

Most developed countries exhibit favorable or improving patterns in the relationship between emissions and economic growth. The average 2018 GDP per capita for these countries is 32,227 in 2011 international dollars, compared to the average of 12,936 dollars of GDP per capita for countries with non-decoupling shapes of the relationship. The total GDP in 2018 for decoupling shapes amount 47,700 billion dollars, whereas that of non-decoupling shapes is 65,573 billion dollars, the two being the 42.11% and the 57.89% of the total GDP, respectively. The total population for countries with decoupling shapes is 1,490 million people, which accounts for 19.79% of the sample population, whereas the population of countries with non-decoupling shapes is 6,043 million people, accounting for 80.21% of the sample. This information leads to a pessimistic outlook for a near and effective decoupling of CO_2_ emissions in world economies. Table 4 shows the behavior of different groups of countries regarding whether the shape of the curves of their members are decoupling or non-decoupling.

**Fig. 5:**
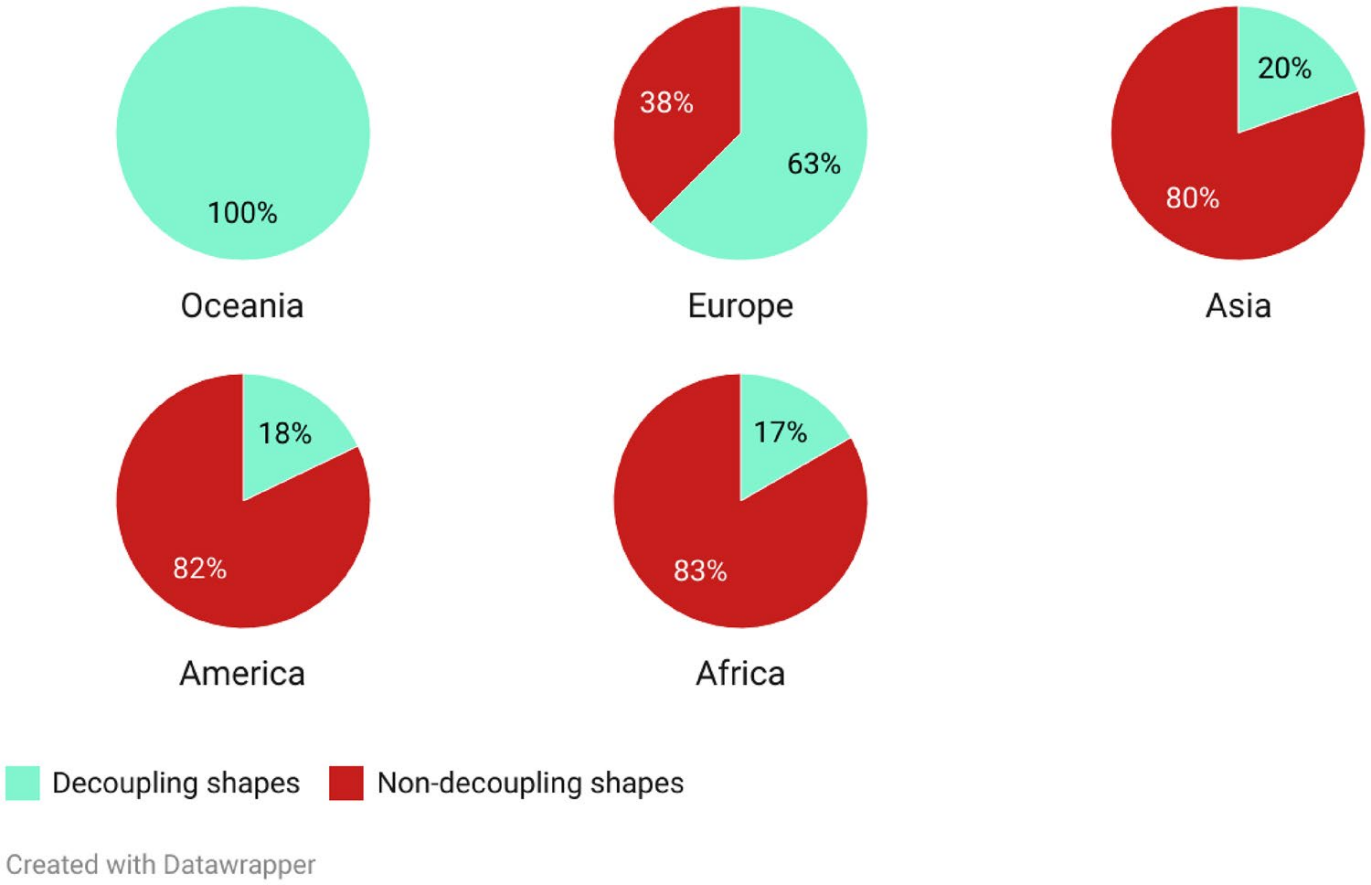
Percentage of decoupling and non-decoupling curve shapes in each continent, ordered from more to less percentage of countries with decoupling curves in each continent. Classification based on the sample of 164 countries. The sample of Oceania only includes two out of fourteen countries: Australia and New Zealand.

To better understand the distribution of decoupling and non-decoupling shapes of the relationship between emissions and economic growth across regions and territories, we have grouped the different countries in different ways. First, Fig. 5 displays the percentage of countries with decoupling and non-decoupling shapes in each continent, ranked in descending order based on the proportion of countries with decoupling shapes. We observe that Oceania and Europe have mostly countries with a decoupling shape, whereas in Asia, America, and Africa, most countries have non-decoupling shapes. The sample of Oceania only includes two out of fourteen countries: Australia and New Zealand.

**Fig. 6:**
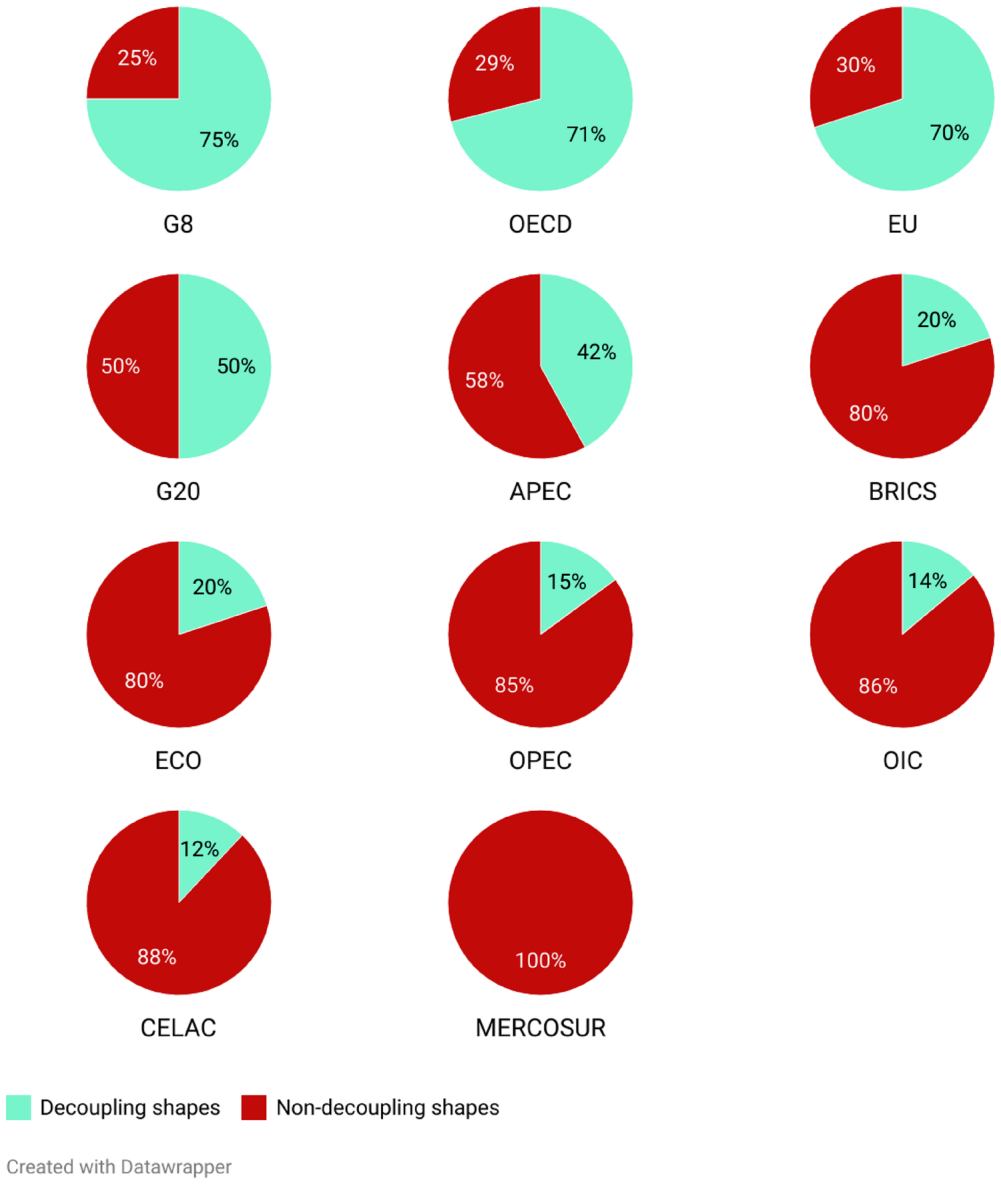
Percentage of the decoupling and non-decoupling shapes in each group of countries. Ordered from more to less percentage of countries with decoupling shapes in each group. EU as a member of G20 has been included in the “decoupling shapes” group for that category of countries. MERCOSUR includes member states and associate states. We have excluded some countries from some groups due to lack of data: Brunei (APEC, OIC), Papua New Guinea (APEC), Somalia (OIC), Sudan (OIC), Maldives (OIC), Guyana (OIC, MERCOSUR, CELAC), Suriname (OIC, MERCOSUR, CELAC), Antigua and Barbuda (CELAC), Bahamas (CELAC), Belize (CELAC), Grenada (CELAC), Saint Kitts and Nevis (CELAC), and Saint Vincent and the Grenadines (CELAC).

We have also classified the different countries into groups based on their membership in international organizations, groups, or internationally accepted classifications. Figure 6 displays the percentage of countries in each group using a multiple pie chart for the 38 countries of the Organisation for Economic Co-operation and Development (OECD), the 27 countries of the European Union (EU) countries, the 5 countries of the emerging economies (BRICS), the 13 countries of the Organization of the Petroleum Exporting Countries (OPEC), the 21 members of the Asia–Pacific Economic Cooperation (APEC), the 10 members of the Economic Cooperation Organization (ECO), the 57 members of the Organization of Islamic Cooperation (OIC), the 9 member states and associate states of the Southern Common Market (MERCOSUR), the 33 members of the Community of Latin American and Caribbean States (CELAC), and the G8 countries and G20 countries. Most countries in the OECD, EU, and G8 show decoupling evolutions of the relationship between emissions and growth. In contrast, BRICS and OPEC countries mostly have non-decoupling trajectories in the relationship. Finally, the G20 has an equal number of countries with proper and non-proper evolutions of the curve.

**Fig. 7:**
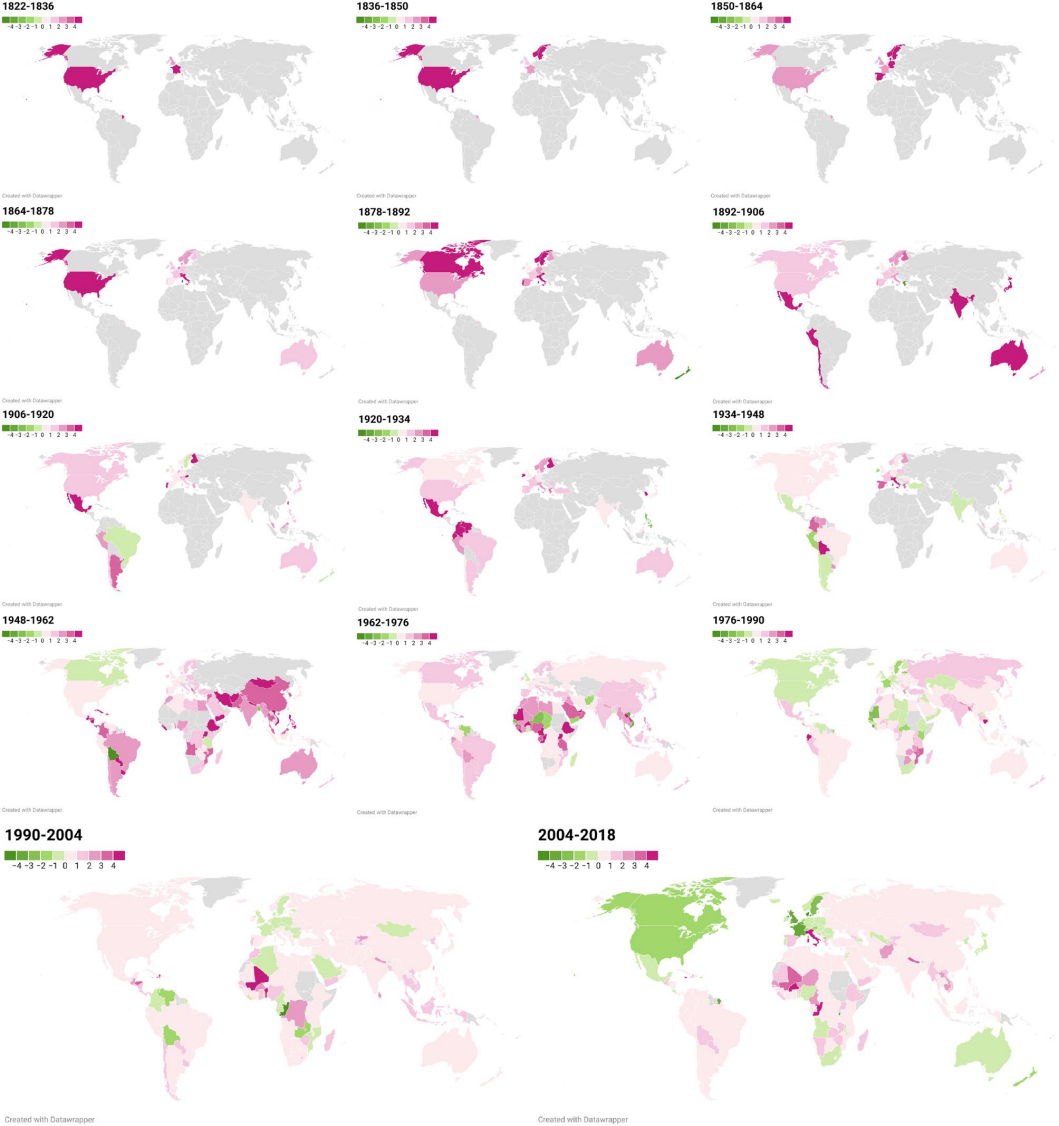
Maps of the evolution of income elasticities in the 164 countries of the sample. The income elasticities of CO_2_ are represented in maps. Red indicates positive elasticities, whereas green indicates negative elasticities between emissions per capita and GDP per capita.

To provide an intuitive and visual representation of the dynamic trends observed in our study, we have summarized the evolution of the elasticities in all countries in Fig. 7, which shows a colored world map for each period. Red represents positive values of elasticities (undesirable situation—positive slope of the relationship), and green shows negative values (desirable situation—negative slope of the relationship). In both cases, the more intense the color, the higher the absolute value.

We observe a transition toward greener maps as we approach more contemporary periods. The intensity of the color also changes, from intense red toward pale tones and from pale greens to more intense ones, although there is no clear pattern, and each country has its own history, as showed in Table 4. We see some countries that transition from red to green but then again to red, like Bolivia, for instance, which shows a N-shaped curve.

**Fig. 8:**
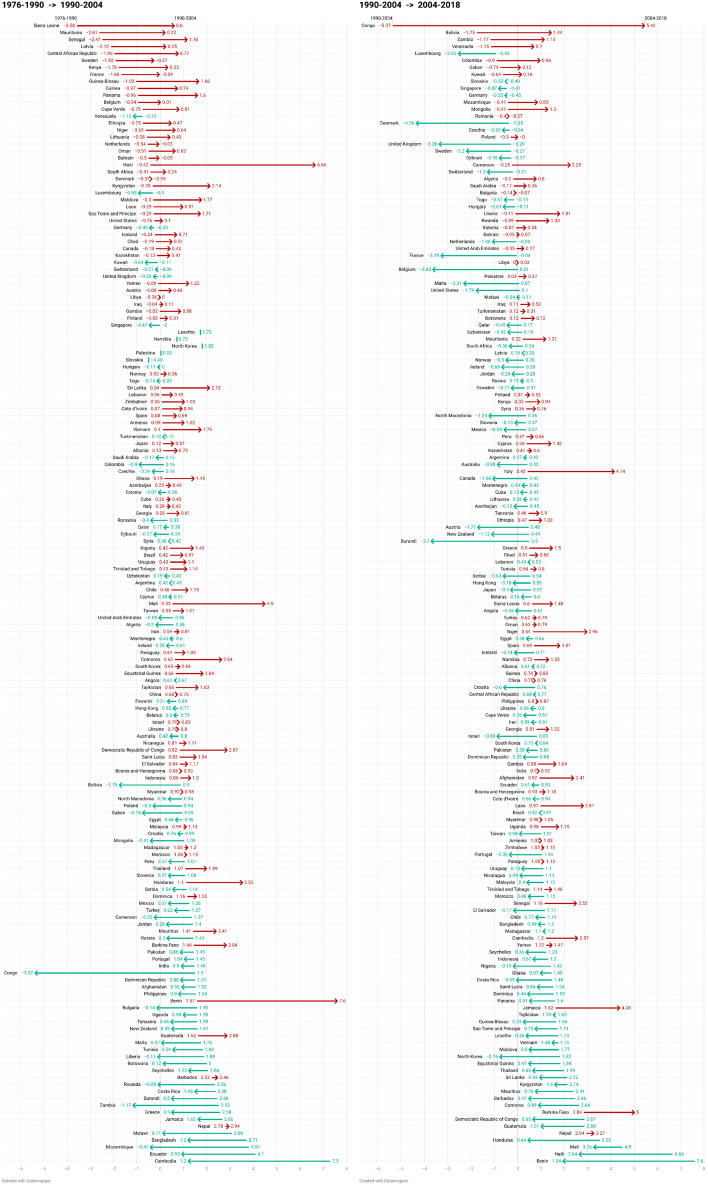
Change in elasticities by country between time frames. Periods 1976–1990 to 1990–2004 and from 1990–2004 to 2004–2018.

We now focus on the last periods of the study. There are 159 country estimates for the period 1976–1990, as shown in Table 3, and we have the full sample of 164 countries from 1990 onward. Furthermore, during these three periods (1976–2018), societies have experienced a growing concern over energy availability, climate change, and the environment in general, demanding stronger environmental policies. Renewable energy sources have become more widespread, triggering energy and sustainable transitions facilitated by supportive legislative measures and policies and increasing environmental awareness. To visualize this transition more clearly, Fig. 8 shows, individually, the change experienced by each country from the period 1976–1990 to the period 1990–2004, and from the period 1990–2004 to the period 2004–2018. Green arrows show a reduction of the elasticity of the country between periods, whereas red arrows indicate an increase of the elasticity between the two periods. Countries are ordered by the lowest value in the first period. Arrows in Fig. 8 show the direction of the evolution of the income elasticity of CO_2_ between periods.

## Discussion

Our analysis offers interesting insights in light of the data and methodology used. Regarding the methodology used, unlike previous studies we avoid problems that have been leading to unreliable parameters, as demonstrated in section "Methods". It is interesting, however, to compare our results with those of other recent studies. The most recent and exhaustive study in terms of the number of countries included is Wang et al.^27^ which estimates the EKC for 29 years (1990–2018) using data from 208 countries or regions worldwide, considering aspects like the role of trade openness, human capital, renewable energy and natural resource rent. This study finds an inverted U-shaped curve at the global level using a classical specification and generalized method of moments (GMM) for dynamic panel data. In 2018, 120 countries did not reach the EKC turning point and 72 countries reached the EKC turning point for per capita income, being this a similar proportion than the results obtained in our study in terms of countries that have reached decoupling versus countries that still have not decoupled their emissions. Similarly, Wang et al.^62^ in another recent research estimate the EKC for 147 countries for the period from 1995 to 2018. They globally substantiate the validity of the EKC hypothesis, but do not provide country level estimates. A more recent study^22^ explores the EKC using a panel data of 38 countries for the period 2002–2020 including other variables like geopolitical risk, natural resource rents, corruption governance, and energy intensity on carbon emissions. They globally find support to the EKC for the selected countries, but no individual results are reported, and their specifications include other factors, so it is not possible to compare our results with theirs. Mohammed et al.^63^ found validity of the EKC for EU-27 countries between 1990 and 2019. However, they use the classical specification with the squared independent variable. On the other side, while we find that 63% of European countries have decoupling shapes, they found a lower percentage (38%).

The studies referred above do not provide estimates of the relationship for individual countries, since they impose in their estimates the same functional form and parameters for the entire set of countries. Golpîra et al.^20^ studied the EKC hypothesis for 37 countries of the OECD over the period from 1960 to 2019. Both the number of countries and the time span are less than in our study, but it is useful to compare their results for individual countries with the results we obtained for the same countries. In their study about 57% of the OECD countries show decoupling shapes while 43% exhibit non-decoupling shapes. Our research, on the other hand, shows that 71% of OECD countries are decoupling versus 29% that have non-decoupling shapes. There are also differences between individual results by country. The existence of multicollinearity due to the use of classical specifications in their estimates lead to unreliable parameter estimates, as demonstrated in section "Unreliable individual EKC estimators with multicollinearity", and it is the main driver of these differences. For BRICS countries, Hasan et al.^64^ found validity of the EKC hypothesis overall and for all the 5 countries analyzed using the classical specification that included a squared independent variable, while we have only found it valid for South Africa. Focusing on studies for individual countries, another recent study^20^ found an inverted U-shape relation between carbon emissions and real GDP in long run for France and Germany for the period 1995–2015. We also find this shape for these countries but using a larger time span with the improved method we propose for correcting multicollinearity. Mahmood et al.^65^ found recently that most EKC studies for China validate the EKC hypothesis while we find a positive slope in the relationship when correcting by multicollinearity. Uche et al.^66^ found an EKC for India for the period 1980–2018 employing the multiple threshold nonlinear ARDL procedure with a squared exogenous variable, while we found an N-shaped curve for this country.

Focusing on the results obtained in this study, it has worrying implications for both regions and individual countries regarding the climate policies carried out over the last decades, considering the urgency required to mitigate climate change. Only in wealthy regions have a majority of countries already decoupled GDP per capita from CO_2_ emissions. However, this represents a small part of the world, and economic growth in most regions will still be associated with higher emissions, leading to greater emissions at the global level. So, the first and most important general implication of these results is that countries must effectively implement, accelerate or rethink this kind of policies, as they may not be working as fast as needed. Given the global nature of the effect of carbon emissions, individual countries have lower incentives than regions or the whole world —many countries have decoupling shapes—. This is why is so important a coordinated and effective action.

## Conclusion

A simple but effective method that eludes multicollinearity and a priori imposing of a specific functional form is proposed in this study to estimate the relationship between CO_2_ emissions and economic growth: segmented-sample regressions. We find that the relationship between CO_2_ per capita and GDP per capita is, on average, still positive, meaning that both variables grow at the same time, although there is a global tendency toward the weakening of this relationship. Only a few countries present a negative elasticity between both variables in any period. When grouping different countries according to income level and taking average elasticities, only the group of wealthiest countries (top 20%) have reversed this association and just during the last period of the analysis (2004–2018). Most countries in Africa (83%), America (82%), and Asia (80%) have non-decoupling shapes, whereas those in Europe and Oceania only represent 38% and 0%, respectively, taking into account that we only consider 2 countries from Oceania (the biggest ones though).

For most countries, the evidence shows that economic growth is still associated with more carbon emissions, which indicates that the decoupling of economic growth and CO_2_ has not yet been achieved on a global scale. Even assuming that the general observed trend of decreasing elasticities over time could eventually lead to turning points in the future in which emissions would start decreasing with economic growth, there would still be positive emissions for most countries. This makes current climate targets, like the Paris Agreement’s target of limiting temperature increase to 1.5ºC or 2ºC above pre-industrial levels, difficult to achieve.

Although the causes and drivers of these tendencies are out of the scope of this research, some important aspects to understand these results are as follows. (1) The tendency to outsource some environmental impacts from developed countries to developing countries due to the stronger environmental regulations in the former^67^ and the tertiarization of economies. Consumption-based emissions can offset this bias if they account for all the impacts along the international supply chain of products and services. (2) The growth of renewables, particularly in some developed countries may be one of the main causes of the appearance of a negative elasticity between both variables, which is positive to reduce CO_2_ emissions^68^. (3) Emissions of international aviation and shipping are not included in any country or region because there is no international agreement on how these emissions should be allocated, representing around 3.45% of total global emissions in 2018^49^. Considering that most commercial aviation is caused by the demand of developed countries and shipping activity feeds in greater measure developed regions, their CO_2_ emissions may be underestimated.

Our proposed method can be applied to future studies to test the EKC hypothesis and inform evidence-based policymaking. In this sense, our study arises some policy implications. The most important one is the need of global efforts to mitigate carbon emissions, not just local/national ones. Territorial emissions in many developed countries appear to be decoupling from CO_2_, but countries need to make efforts not just to reduce their emissions but also to help reducing emissions in other countries, like developing ones, with a fair and global justice perspective. Climate change is a global problem with global consequences and, in terms of decoupling, our research suggests a gap between developed and developing countries, or sometimes different velocities in decoupling. The articulation of the different potential mechanisms to achieve decoupling is out of the scope of this research, but dialogue, common understanding and cooperation between countries suppose the first steps in solving complex global issues. National efforts are important, but climate policies need to have a global perspective to be effective, given that the climate and the atmosphere have characteristics of a pure public good. A second policy implication is that policies should be oriented to global decoupling but also, and most important, towards achieving global absolute reductions of CO_2_ emissions. Decoupling of some countries or groups of countries in not enough and highlights the importance of reading the results of this research with a global perspective. One country may reach the wrong conclusion from this research that no more climate policy efforts are needed if they are already decoupling. All the contrary, their territorial emissions may be decoupling from GDP but, at the same time, causing other territories to emit more CO_2_ due to other complex economic mechanisms mentioned in this article. From a public policy perspective there is no relieve in one territory from being decoupling if globally the other countries are not. Although several countries are decoupling, CO_2_ emissions are still globally growing. The relevant aspect in climate policy is the achievement of global absolute reductions of CO_2_ emissions. Following the last assessment report of the IPCC^69^, they need to be reduced fast to avoid the worst consequences of climate change.

Further research should focus not just on monitoring this relationship in the future but also on estimating this relationship for other environmental impacts, not just CO_2_ emissions and climate change, understanding the links between impacts in an energy/sustainable transition, and studying its relationship with economic growth. It is important to consider that economic growth may be the cause of other environmental problems, beyond CO_2_ emissions, not considered in this study. Some studies, for instance, link the deployment of renewable energy sources with the use of more other natural resources, some of them critical materials^70^. Our results are concerning and emphasize the need to accelerate changes toward a low-carbon economy and implement effective and global policies that incentivize emissions reductions.

## Supplementary Information

Supplementary Information.

## Data Availability

Data is available upon reasonable request. Please contact the corresponding author.
