# Author Correction: World economies’ progress in decoupling from CO_2_ emissions

**DOI:** 10.1038/s41598-024-75404-2

**Published:** 2024-11-08

**Authors:** Jaume Freire-González, Emilio Padilla Rosa, Josep Ll. Raymond

**Affiliations:** 1 Institute for Economic Analysis (CSIC) and Barcelona School of Economics, 08193 Bellaterra, Barcelona, Spain; 2 Department of Applied Economics, Univ. Autonoma de Barcelona, 08193 Bellaterra, Spain; 3 Department of Economics and Economic History, Univ. Autonoma de Barcelona, 08193 Bellaterra, Spain

Correction to: *Scientific Reports* 10.1038/s41598-024-71101-2, Published online 03 September 2024

The original version of this Article contained errors in Tables 1 and 2, where the p-values were not in parentheses.

The original Article has been corrected.

