# Supplementary material for: World economies’ progress in decoupling from CO2 emissions
Source: Sci Rep. 2024 Sep 3;14:20480. doi: 10.1038/s41598-024-71101-2 (PMC11372050; doi:10.1038/s41598-024-71101-2)
Supplement: Supplementary file 1 — Supplementary Information. [file 41598_2024_71101_MOESM1_ESM.pdf]

Appendix I. Descriptive statistics of the main variables used in the analysis.

Table A1. Descriptive statistics of production-based CO_2 emissions per capita and GDP per capita for the period 1822–2018, ordered by 2018 GDP per capita.

	CO_2 per capita					GDP per capita				
Country	Sample (nº years)	Mean	Standard deviation	Minimum	Maximum	Sample (nº years)	Mean	Standard deviation	Minimum	Maximum
Qatar	73	44.88	24.21	4.02	120.33	69	60,018	42,610	11,823	166,150
Norway	190	3.57	3.30	0.00	9.70	190	15,239	22,158	1,358	84,584
United Arab Emirates	63	28.64	18.09	0.08	80.97	67	41,466	17,985	19,483	80,404
Singapore	72	8.50	4.70	0.25	17.47	98	17,880	20,602	1,418	70,537
Ireland	111	5.63	3.52	0.05	12.43	101	17,327	17,351	1,402	65,016
Kuwait	74	29.03	41.80	0.74	367.93	69	40,564	20,281	9,783	79,469
Switzerland	164	3.11	2.25	0.06	7.33	168	17,392	16,807	2,261	62,479
Luxembourg	77	25.78	9.92	0.09	41.05	69	33,049	15,738	12,304	58,554
United States	222	10.02	8.03	0.04	23.08	199	16,222	14,976	2,678	54,617
Hong Kong	78	3.46	2.18	0.01	6.41	72	21,067	15,028	920	49,947
Australia	162	7.74	6.41	0.18	19.21	199	13,276	12,601	832	49,584
Saudi Arabia	84	10.07	6.90	0.00	20.73	72	19,237	13,312	797	49,519
Netherlands	176	5.54	3.76	0.95	13.32	199	12,674	12,448	2,952	47,325
Germany	222	5.89	4.72	0.03	14.35	170	12,759	12,608	1,576	46,877
Denmark	179	4.92	4.12	0.10	14.24	199	12,669	13,120	2,028	46,529
Sweden	184	3.52	3.08	0.01	11.50	199	11,422	12,674	1,411	45,601
Canada	223	7.12	6.75	0.00	18.47	154	15,536	13,166	1,430	45,076
Taiwan	126	3.65	4.21	0.00	12.20	111	10,380	12,867	958	43,907
Iceland	83	7.25	3.28	0.03	11.96	69	24,513	10,720	8,091	43,392
Austria	204	3.81	2.99	0.05	9.61	154	13,285	12,719	1,942	43,241
Bahrain	89	19.48	8.59	0.09	40.27	69	13,348	12,559	3,288	42,118
France	214	3.74	2.87	0.07	10.40	199	11,345	11,763	1,806	40,164
Belgium	193	7.67	3.64	1.14	14.25	174	12,958	11,231	2,349	39,689
Finland	162	4.05	4.72	0.02	13.94	161	11,516	12,087	1,315	40,130
Japan	154	3.81	3.84	0.00	10.30	139	12,566	12,864	1,320	38,549
United Kingdom	227	7.47	3.21	1.01	11.82	199	12,498	9,945	3,298	38,237
South Korea	113	3.61	4.55	0.00	12.97	108	9,161	11,344	816	37,897
Oman	58	8.89	5.56	0.02	17.92	69	15,135	14,120	1,061	47,684
New Zealand	144	4.74	2.21	0.77	9.06	152	14,190	8,685	809	35,344
Italy	162	2.82	3.04	0.00	8.67	199	9,864	10,736	2,352	36,116
Israel	92	5.04	3.31	0.06	10.10	69	18,335	8,116	4,497	32,970
Spain	192	2.08	2.51	0.00	8.47	173	8,539	9,626	1,596	33,244
Malta	72	3.79	2.24	0.74	7.65	69	11,979	8,985	1,681	31,747
Czechia	162	7.43	5.52	0.03	18.39	49	17,223	6,547	8,748	30,955
Slovenia	126	3.62	3.26	0.00	8.99	67	17,175	7,171	3,978	28,700
Poland	222	3.90	3.87	0.04	13.04	89	8,982	6,395	818	27,348
Seychelles	59	2.81	1.79	0.14	5.83	69	9,250	6,970	2,278	27,101
Slovakia	162	5.63	3.31	0.03	12.29	34	16,829	5,300	9,776	26,889
Portugal	152	1.82	2.01	0.01	6.68	187	6,625	7,780	1,410	26,856
Estonia	170	7.06	7.97	0.00	25.45	40	18,519	4,152	11,963	26,782
Lithuania	170	2.85	3.29	0.00	11.02	40	15,045	5,142	7,589	26,639
Hungary	189	2.73	2.66	0.01	8.56	98	9,121	6,023	1,744	25,616
Kazakhstan	170	6.23	6.22	0.00	17.90	40	14,370	5,517	7,481	25,014
Russia	170	5.16	5.61	0.00	17.13	59	13,099	5,613	5,552	24,808
Latvia	170	2.41	2.79	0.00	9.71	40	15,108	4,390	8,199	24,210
Malaysia	131	2.13	2.63	0.00	8.24	117	6,065	6,017	831	23,879
Greece	141	2.87	3.50	0.00	10.33	186	6,944	7,519	1,338	28,728
Turkmenistan	170	4.36	4.35	0.00	13.09	40	9,066	6,042	2,749	23,477
Trinidad and Tobago	113	13.06	9.50	0.08	33.30	69	15,922	6,204	5,706	27,786
Cyprus	72	4.61	2.38	0.54	7.98	69	11,966	7,385	2,786	24,314
Chile	127	1.91	1.30	0.18	4.83	199	5,631	5,289	787	22,075
Mauritius	72	1.43	1.13	0.21	3.50	69	9,751	5,112	3,611	21,210
Panama	74	1.61	0.70	0.03	3.17	113	5,983	4,555	2,348	20,656
Croatia	126	2.00	1.88	0.00	5.64	67	11,190	4,918	2,701	20,425
Uruguay	90	1.40	0.74	0.03	2.55	151	7,247	4,195	1,948	20,006
Turkey	157	1.21	1.57	0.01	5.26	99	6,724	5,813	971	20,564
Romania	164	2.60	2.81	0.00	9.37	104	4,747	5,301	363	19,699
Montenegro	126	1.42	1.32	0.00	4.13	67	6,905	4,562	1,539	18,973
Argentina	135	2.25	1.44	0.27	4.67	123	9,796	4,457	1,600	20,134
Bulgaria	141	3.35	3.50	0.00	10.30	97	7,038	4,572	1,329	18,572
Belarus	170	3.13	3.48	0.00	11.90	40	12,127	3,788	6,822	18,657
Gabon	63	4.14	2.59	0.04	10.61	69	8,323	4,270	2,601	17,354
Iran	112	3.32	2.43	0.00	8.52	72	8,003	4,705	877	17,715
Lebanon	90	2.08	1.43	0.00	4.57	72	7,616	4,398	1,083	19,604
Mexico	131	2.29	1.60	0.04	6.07	128	6,254	4,592	1,017	16,180
Thailand	88	1.41	1.51	0.00	4.17	75	6,074	4,581	919	15,803
Azerbaijan	170	2.43	2.43	0.00	7.61	40	8,945	4,672	2,939	16,651
Georgia	170	0.90	1.02	0.00	2.99	40	9,826	3,657	3,563	15,636
Libya	72	7.14	4.26	0.13	19.07	69	8,017	6,542	527	29,658
Dominican Republic	75	1.28	0.80	0.00	2.61	69	5,148	3,677	1,617	15,350
Equatorial Guinea	72	1.98	3.11	0.05	12.06	69	7,447	9,246	743	29,446
Botswana	50	1.72	0.78	0.04	3.06	69	4,883	4,649	509	14,622
Costa Rica	72	1.03	0.47	0.30	1.79	99	5,952	3,485	2,049	14,549
Brazil	121	0.92	0.81	0.09	2.74	151	4,230	4,269	846	15,940
Algeria	106	1.61	1.53	0.00	4.20	72	5,787	3,803	686	14,247
Serbia	126	3.00	2.65	0.01	7.80	67	7,780	3,160	2,017	13,448
Colombia	101	1.12	0.64	0.00	2.10	123	4,919	3,443	774	13,229
Mongolia	72	4.29	3.74	0.37	15.28	69	3,257	3,570	675	13,126
North Macedonia	126	2.77	2.34	0.00	7.09	67	7,561	2,877	2,085	13,108

China	115	1.76	2.29	0.04	8.05	89	3,220	3,320	803	12,809
Iraq	94	2.48	1.39	0.04	5.34	72	5,743	3,307	877	13,689
Barbados	83	2.57	1.84	0.02	5.91	69	10,118	3,253	3,397	13,614
Bosnia and Herzegovina	126	2.03	1.95	0.00	6.49	67	5,774	2,670	1,858	12,143
Peru	138	0.84	0.50	0.00	1.96	127	4,427	2,699	586	11,996
Albania	89	1.23	0.74	0.01	2.74	75	4,193	2,876	707	11,821
Egypt	111	0.90	0.81	0.00	2.56	72	4,418	3,208	959	11,778
Sri Lanka	72	0.41	0.26	0.16	1.07	151	2,912	2,315	857	11,745
South Africa	138	5.08	2.93	0.00	9.79	71	6,992	2,432	867	11,848
Armenia	170	0.88	0.88	0.00	2.76	40	8,085	2,438	3,804	11,738
Indonesia	133	0.59	0.64	0.00	2.45	192	2,301	2,237	750	11,516
Tunisia	94	1.10	0.89	0.00	2.62	72	5,174	3,035	684	10,921
Uzbekistan	170	2.23	2.11	0.00	5.68	40	6,588	1,638	4,464	10,381
Venezuela	111	4.50	2.92	0.00	16.47	151	7,648	6,006	949	20,933
Ecuador	105	1.06	0.87	0.01	2.74	120	4,240	2,822	753	10,826
Paraguay	72	0.54	0.34	0.03	1.32	80	4,611	2,229	2,202	10,289
Jordan	72	2.13	1.01	0.34	3.60	72	6,171	2,692	873	11,825
Saint Lucia	72	1.32	0.99	0.04	2.89	69	4,634	2,968	1,121	9,746
Ukraine	170	3.92	4.41	0.00	14.60	40	8,314	1,911	4,631	10,846
Namibia	31	1.24	0.31	0.75	1.78	69	6,382	1,526	3,611	9,981
Dominica	71	1.01	0.87	0.07	2.66	69	4,070	3,005	995	10,082
El Salvador	72	0.62	0.33	0.12	1.18	99	3,505	2,037	1,161	8,724
Philippines	112	0.45	0.41	0.00	1.32	114	3,149	1,552	928	8,404
Morocco	94	0.72	0.58	0.00	1.90	72	4,008	1,885	686	8,350
Cuba	81	2.03	0.94	0.02	3.48	118	3,315	1,621	888	8,170
Guatemala	81	0.53	0.31	0.00	1.16	99	4,444	1,576	1,937	7,570
Eswatini	70	0.68	0.37	0.01	1.25	69	3,491	2,206	617	7,559
Moldova	170	2.23	2.75	0.00	9.32	40	6,107	2,549	2,909	10,146
Vietnam	130	0.50	0.71	0.02	3.56	72	2,261	1,582	821	6,969
Jamaica	72	2.71	1.16	0.19	4.38	82	4,964	2,032	841	7,421
Cape Verde	72	0.47	0.36	0.06	1.17	69	2,514	1,904	794	6,797
Laos	67	0.41	0.77	0.01	2.89	69	1,945	1,540	780	6,568
Bolivia	94	0.74	0.64	0.00	2.05	119	2,965	1,213	1,350	6,522
India	153	0.38	0.49	0.00	1.93	142	1,607	1,160	828	6,454
Myanmar	94	0.19	0.13	0.00	0.68	81	1,764	1,299	693	6,030
Angola	72	0.54	0.33	0.04	1.25	69	2,321	1,650	766	6,287
Pakistan	76	0.49	0.26	0.02	1.00	69	2,461	1,228	1,012	5,516
Congo	63	0.52	0.35	0.12	1.32	69	3,387	1,354	1,675	6,081
Nigeria	107	0.34	0.32	0.00	1.02	69	2,366	1,396	1,026	5,700
Palestine	32	0.52	0.13	0.30	0.77	71	3,876	1,194	1,667	6,106
Kyrgyzstan	170	1.53	1.72	0.00	5.39	40	4,314	1,036	2,728	5,869
Honduras	72	0.61	0.29	0.19	1.13	99	2,574	844	1,420	4,634
Nicaragua	79	0.53	0.25	0.00	0.93	99	2,739	923	1,316	4,848
Bangladesh	76	0.16	0.15	0.00	0.55	69	1,453	861	806	4,194
Tajikistan	170	0.84	0.95	0.00	2.85	40	3,440	1,471	1,488	6,458
Cote d'Ivoire	64	0.41	0.15	0.12	0.80	69	2,591	543	1,734	4,024
Ghana	72	0.30	0.12	0.13	0.65	71	2,124	588	507	3,851
Cambodia	67	0.20	0.27	0.00	1.15	69	1,612	679	784	3,716
Sao Tome and Principe	71	0.35	0.18	0.06	0.62	69	2,234	663	1,094	3,608
Kenya	72	0.29	0.05	0.16	0.39	69	1,792	589	1,081	3,473
Zambia	72	0.64	0.43	0.18	1.61	69	1,777	669	1,161	3,419
Mauritania	63	0.49	0.32	0.04	1.75	69	1,812	603	1,037	3,165
Cameroon	72	0.24	0.17	0.03	0.71	69	1,978	515	1,212	3,037
Syria	91	1.50	1.21	0.00	3.34	72	6,691	2,505	1,084	10,828
Nepal	72	0.09	0.13	0.00	0.52	72	1,332	528	639	2,843
Tanzania	72	0.12	0.05	0.06	0.22	69	1,158	571	703	2,790
Djibouti	72	0.63	0.25	0.18	1.21	69	3,382	1,478	1,655	5,951
Senegal	64	0.43	0.16	0.02	0.81	69	2,219	151	1,952	2,748
Lesotho	32	1.02	0.16	0.82	1.51	69	1,571	496	791	2,521
Yemen	72	0.59	0.29	0.01	1.79	69	3,032	1,079	1,472	4,775
Benin	64	0.22	0.18	0.04	0.62	69	1,573	287	1,132	2,235
Uganda	72	0.07	0.03	0.02	0.14	69	1,229	340	826	2,086
Chad	63	0.06	0.03	0.01	0.14	69	992	428	533	2,331
Afghanistan	73	0.15	0.10	0.00	0.40	69	1,328	365	512	1,997
Rwanda	72	0.06	0.04	0.00	0.14	69	1,272	232	630	1,834
Comoros	63	0.17	0.08	0.05	0.39	69	1,097	360	488	1,824
Ethiopia	77	0.05	0.04	0.00	0.16	69	955	227	704	1,761
Haiti	72	0.14	0.07	0.02	0.31	74	1,622	141	1,231	2,003
Gambia	72	0.15	0.07	0.03	0.26	69	1,288	192	867	1,611
North Korea	113	2.49	2.51	0.00	10.21	32	1,687	336	731	2,415
Burkina Faso	64	0.08	0.07	0.00	0.26	69	1,137	163	785	1,536
Guinea	64	0.19	0.05	0.05	0.36	69	835	265	451	1,517
Zimbabwe	119	1.14	0.45	0.10	1.98	69	1,823	382	1,142	2,423
Togo	72	0.20	0.11	0.02	0.50	69	1,183	216	729	1,566
Guinea-Bissau	72	0.13	0.06	0.01	0.24	69	1,171	254	487	1,527
Mali	63	0.08	0.05	0.01	0.19	69	996	233	572	1,381
Madagascar	79	0.10	0.04	0.00	0.22	69	1,511	316	1,074	2,009
Sierra Leone	72	0.13	0.07	0.04	0.40	69	1,282	264	680	1,614
Malawi	72	0.09	0.02	0.06	0.14	69	860	210	493	1,229
Mozambique	95	0.15	0.12	0.00	0.42	69	1,772	679	838	3,302
Democratic Republic of Congo	102	0.08	0.07	0.00	0.25	69	971	309	439	1,422
Niger	64	0.07	0.04	0.01	0.14	69	1,035	294	643	1,597
Liberia	72	0.35	0.30	0.05	1.06	69	2,436	1,443	366	4,476
Central African Republic	63	0.06	0.02	0.02	0.10	69	1,114	202	610	1,396
Burundi	72	0.03	0.02	0.00	0.06	69	818	150	624	1,129

Source: own elaboration.

Appendix II. Results of estimates by country and period.

Table A2. Estimated income elasticities of CO_2 by country and period, ordered by 2018 GDP per capita.

Country	1822-1836	1836-1850	1850-1864	1864-1878	1878-1892	1892-1906	1906-1920	1920-1934	1934-1948	1948-1962	1962-1976	1976-1990	1990-2004	2004-2018
Qatar										-1.49	-0.82	0.38	0.17	-0.45
Norway		15.50	4.54	2.50	4.11	2.06	0.52	2.25	1.86	0.58	1.57	0.02	0.26	-0.50
United Arab Emirates											15.97	0.56	-0.05	0.27
Singapore										-1.66	2.66	0.00	-0.47	-0.82
Ireland								5.13	-1.09	1.36	1.08	0.61	0.28	-0.68
Kuwait										-3.83	1.04	-0.11	-0.64	0.16
Switzerland				4.15	1.55	1.70	2.46	1.25	3.02	1.46	1.10	-0.09	-0.21	-1.30
Luxembourg										0.94	0.19	-0.30	-0.93	-2.55
United States	4.94	6.80	2.25	6.29	2.66	1.79	1.35	1.20	0.71	0.24	0.96	-0.26	0.10	-1.79
Hong Kong										1.09	1.19	0.77	0.55	-0.18
Australia				1.44	2.51	4.04	1.01	1.57	0.77	2.06	0.89	0.80	0.42	-0.88
Saudi Arabia										1.02	3.18	0.15	-0.17	0.36
Netherlands			1.49	3.49	1.72	1.77	2.02	1.56	1.22	0.71	1.17	-0.54	-0.05	-1.08
Germany			4.15	1.73	2.42	1.66	0.56	0.40	0.80	0.67	0.25	-0.25	-0.45	-0.55
Denmark			6.29	2.84	2.78	2.04	1.31	1.74	0.13	1.21	0.92	-0.37	-0.35	-4.36
Sweden		10.02	8.91	2.82	4.76	2.07	-0.36	2.25	0.32	1.27	1.03	-1.92	-0.27	-2.20
Canada					7.75	1.71	1.56	0.58	0.71	-0.16	1.06	-0.18	0.42	-1.06
Taiwan							5.40	0.17		1.57	0.96	0.55	1.01	0.08
Iceland										1.06	0.28	-0.24	0.71	-0.14
Austria					4.52	2.47	6.50	0.82	-0.32	0.38	0.89	-0.08	0.48	-1.71
Bahrain										-1.16	3.48	-0.50	-0.05	0.07
France	6.12	2.65	2.77	1.23	1.72	1.81	0.55	1.10	1.15	0.61	0.72	-1.66	-0.04	-3.29
Belgium			1.76	1.46	1.30	1.78	1.60	2.15	2.09	0.26	0.45	-0.94	0.01	-3.63
Finland				1.80	2.71	3.92	6.92	5.53	2.47	1.61	1.72	-0.02	0.31	0.52
Japan						6.09	1.79	0.56	1.46	0.97	1.16	0.12	0.57	-0.30
United Kingdom	2.07	1.89	2.04	1.76	0.88	0.96	0.25	0.51	0.01	0.46	-0.03	-0.09	-0.29	-3.38
South Korea								5.16	-0.43	3.74	1.23	0.65	0.84	0.75
Oman											3.22	-0.51	0.63	0.79
New Zealand					-4.11	2.08	-0.35	0.96	0.40	1.04	1.06	1.61	0.49	-1.12
Italy				14.81	8.69	2.70	1.05	2.24	4.73	1.63	1.66	0.28	0.42	4.14
Israel										1.05	0.59	0.79	0.83	-0.88
Spain			10.54	0.89	2.32	1.47	0.09	1.78	3.22	0.87	1.50	0.08	0.69	1.81
Malta										0.59	0.70	1.76	0.07	-2.31
Czechia												0.16	-0.34	-0.65
Slovenia										1.08	1.07	1.08	0.37	-0.13
Poland									1.72	1.34	0.77	0.94	-0.30	0.00
Seychelles											5.81	2.06	1.23	0.36
Slovakia													-0.49	-0.53
Portugal					7.46	2.32	10.60	1.37	0.24	1.08	1.06	1.45	1.04	-0.35
Estonia												0.26	-0.07	0.34
Lithuania												-0.58	0.43	0.26
Hungary									0.47	1.78	0.82	0.00	-0.11	-0.61
Kazakhstan												-0.13	0.41	0.60
Russia											0.89	1.43	0.30	0.13
Latvia												-2.12	0.25	0.19
Malaysia							2.89	0.11	1.16	3.15	1.86	0.99	1.13	0.40
Greece						-5.67	2.92	3.15	3.42	2.17	1.48	2.58	0.50	1.50
Turkmenistan												0.12	0.12	0.31
Trinidad and Tobago										0.42	3.61	0.43	1.14	1.46
Cyprus										3.36	1.29	0.51	0.38	1.42
Chile						7.31	1.10	0.52	-0.19	2.31	1.17	0.48	1.19	0.77
Mauritius										-0.11	0.43	1.41	2.41	0.76
Panama										4.16	1.67	-0.96	1.60	0.51
Croatia											1.15	0.99	0.76	-0.60
Uruguay									2.27	9.99	1.16	0.43	1.10	0.18
Turkey								1.55	-0.67	1.12	1.41	1.27	0.62	0.79
Romania								1.07	0.43	1.41	0.87	0.33	-0.40	-0.37
Montenegro										1.09	1.17	0.60	0.43	0.19
Argentina							3.01	1.99	-0.76	2.22	1.22	0.45	0.42	0.27
Bulgaria									1.74	1.72	1.25	1.59	-0.14	-0.07
Belarus												0.79	0.60	0.16
Gabon											3.51	0.95	-0.74	0.12
Iran										4.10	0.92	0.59	0.81	0.59
Lebanon										0.01	0.27	0.06	0.53	0.45
Mexico						4.55	5.81	5.23	-0.45	0.96	1.29	1.26	0.37	-0.59
Thailand										3.20	2.34	1.07	1.99	0.65
Azerbaijan												0.25	0.45	-0.15
Georgia												0.28	0.81	1.32
Libya										1.42	2.11	-0.06	0.00	0.03
Dominican Republic										3.34	2.49	1.51	0.88	0.25
Equatorial Guinea										1.13	1.53	0.66	1.84	0.47
Botswana												2.00	0.12	0.72
Costa Rica										0.16	1.95	2.38	1.48	-0.05
Brazil							-0.19	1.54	0.46	2.06	1.09	0.42	0.97	0.92
Algeria										0.57	2.99	0.58	-0.20	0.80
Serbia										0.92	1.17	1.14	0.54	-0.63
Colombia								5.89	3.89	3.68	0.73	0.16	-0.90	0.96
Mongolia										5.49	1.45	1.05	-0.41	1.30
North Macedonia										1.34	0.96	0.94	0.36	-1.24
China										3.43	1.57	0.68	0.75	0.76
Iraq										1.68	2.05	-0.04	0.11	0.52

Barbados					1.56	1.55	2.22	2.46	0.47
Bosnia and Herzegovina					1.16	1.27	0.86	0.93	1.18
Peru	5.49	2.59	2.30	-1.26	0.26	1.66	1.07	0.37	0.66
Albania					4.69	1.58	0.13	0.72	0.61
Egypt					2.02	0.59	0.96	0.66	0.38
Sri Lanka					0.41	0.29	0.04	2.12	0.93
South Africa					1.14	0.70	-0.41	0.24	-0.36
Armenia							0.09	1.02	1.03
Indonesia					0.85	1.38	0.86	1.30	0.67
Tunisia					0.44	1.36	1.83	0.54	0.80
Uzbekistan							0.43	0.19	-0.42
Venezuela			5.21	2.13	0.38	-1.01	-0.75	-1.15	0.70
Ecuador			21.30	0.10	0.27	2.49	4.10	0.93	0.61
Paraguay					8.32	1.41	0.61	1.05	1.13
Jordan					2.86	0.47	1.40	0.28	-0.24
Saint Lucia					2.20	3.37	0.83	1.54	0.84
Ukraine							0.79	0.80	0.56
Namibia								0.72	1.33
Dominica					1.24	1.16	1.16	1.55	0.44
El Salvador					1.78	1.73	0.84	1.17	-0.17
Philippines		1.10	-2.21	-0.11	8.56	2.38	1.54	0.80	0.87
Morocco					2.35	2.68	1.06	1.15	0.46
Cuba					15.75	1.52	0.26	0.43	0.13
Guatemala					4.84	1.16	1.62	2.88	1.01
Eswatini					2.01	1.40	0.69	0.31	-0.27
Moldova							-0.30	1.77	0.50
Vietnam					7.43	-3.01	0.10	1.75	1.48
Jamaica					2.09	2.25	2.66	1.62	4.38
Cape Verde					-3.51	1.48	-0.75	0.81	0.26
Laos						5.52	-0.29	0.97	2.81
Bolivia				4.72	-4.10	2.30	0.90	-1.75	1.44
India	7.32	0.23	0.33	-0.47	2.48	1.37	1.48	0.90	0.92
Myanmar					2.83	2.48	0.92	0.98	1.05
Angola					3.85	0.17	0.67	0.61	-0.08
Pakistan					3.13	0.22	1.45	0.86	0.38
Congo						4.65	1.50	-5.37	5.44
Nigeria					2.45	3.03	0.42	1.43	-0.19
Palestine								0.03	0.37
Kyrgyzstan							-0.33	2.14	1.60
Honduras					3.31	2.68	1.10	3.53	0.44
Nicaragua					10.81	1.99	0.81	1.11	0.09
Bangladesh					-2.53	-0.66	3.71	1.20	0.89
Tajikistan							0.68	1.63	1.53
Cote d'Ivoire						2.71	0.07	0.94	0.66
Ghana					0.98	-0.03	0.19	1.45	0.97
Cambodia						2.35	7.30	1.20	2.57
Sao Tome and Principe					2.57	1.02	-0.29	1.71	0.75
Kenya					1.69	0.40	-1.78	0.33	0.94
Zambia					0.48	0.01	2.53	-1.17	1.15
Mauritania						4.82	-2.61	0.22	1.21
Cameroon					1.64	7.29	1.37	-0.25	2.25
Syria					3.14	1.17	0.42	0.36	0.76
Nepal					7.37	2.13	2.78	2.94	3.27
Tanzania					-0.89	3.54	1.59	0.46	0.90
Djibouti					2.19	1.05	0.39	-0.27	-0.78
Senegal						-2.83	-2.47	1.16	2.52
Lesotho								1.73	0.36
Yemen					39.84	-1.61	-0.09	1.22	1.47
Benin						3.83	1.57	7.60	1.94
Uganda					6.76	4.38	1.59	0.98	1.75
Chad						-1.99	-0.19	0.51	0.86
Afghanistan					10.07	-1.75	1.52	0.92	2.41
Rwanda					6.58	2.37	2.36	-0.09	1.33
Comoros						1.63	0.63	2.64	0.89
Ethiopia					10.02	5.86	-0.75	0.47	1.03
Haiti					-0.56	2.49	-0.42	6.66	2.64
Gambia					1.18	1.35	-0.02	0.88	1.64
North Korea								1.82	-0.76
Burkina Faso						7.66	1.44	2.84	5.00
Guinea						3.14	-0.97	0.74	0.83
Zimbabwe					0.59	0.75	0.06	1.03	1.13
Togo					3.29	2.54	0.03	-0.14	-0.57
Guinea-Bissau					2.19	1.36	-1.03	1.66	0.24
Mali						2.96	0.53	4.50	3.26
Madagascar					1.03	-0.05	1.05	1.20	1.10
Sierra Leone					3.50	3.19	-3.58	0.60	1.48
Malawi					1.41	0.50	3.08	0.11	-0.04
Mozambique					3.20	1.25	3.81	-0.41	0.85
Democratic Republic of Congo					1.74	0.64	0.82	2.87	0.65
Niger						-2.09	-0.63	0.64	2.96
Liberia					6.76	4.57	1.88	-0.11	1.81
Central African Republic						0.06	-1.96	0.77	0.68
Burundi					0.77	1.25	2.46	0.50	-3.70

Source: own elaboration.

Appendix III. Relationship between CO2 emissions per capita and GDP per capita, by country.

Figure A3. Curves representing the relationship between CO2 emissions per capita and GDP per capita by country, in income elasticities of CO_2, ordered by deciles and 2018 GDP per capita.

Decile 10:

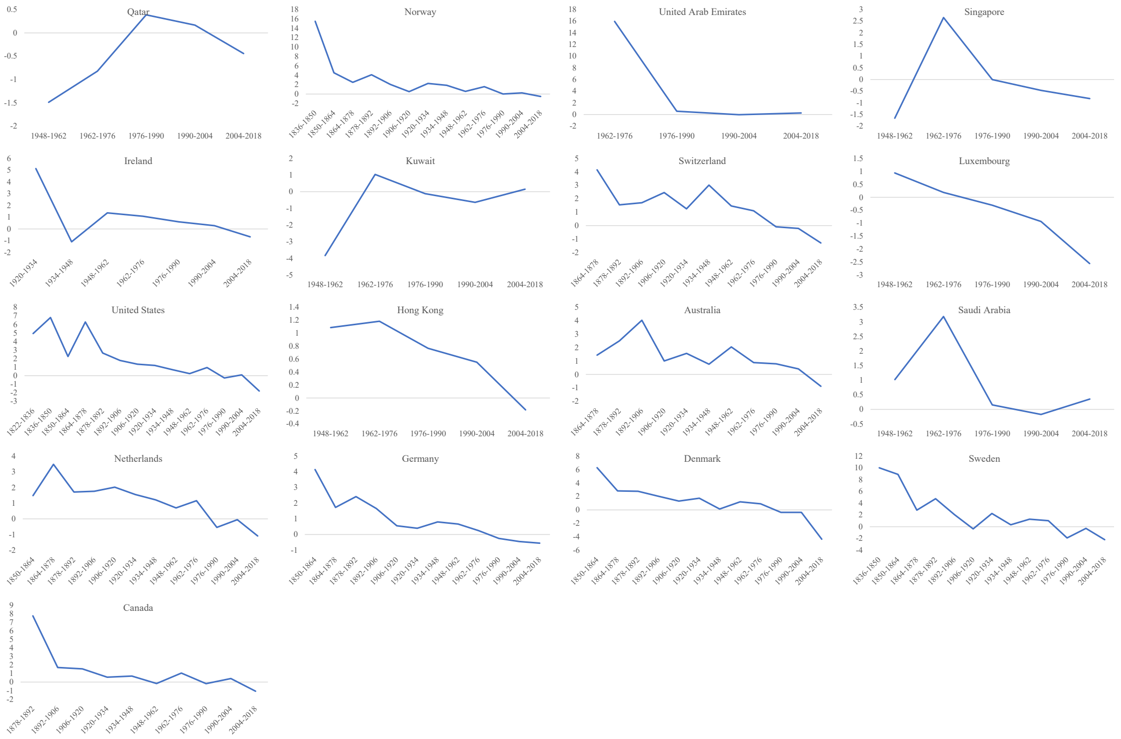

Decile 9:

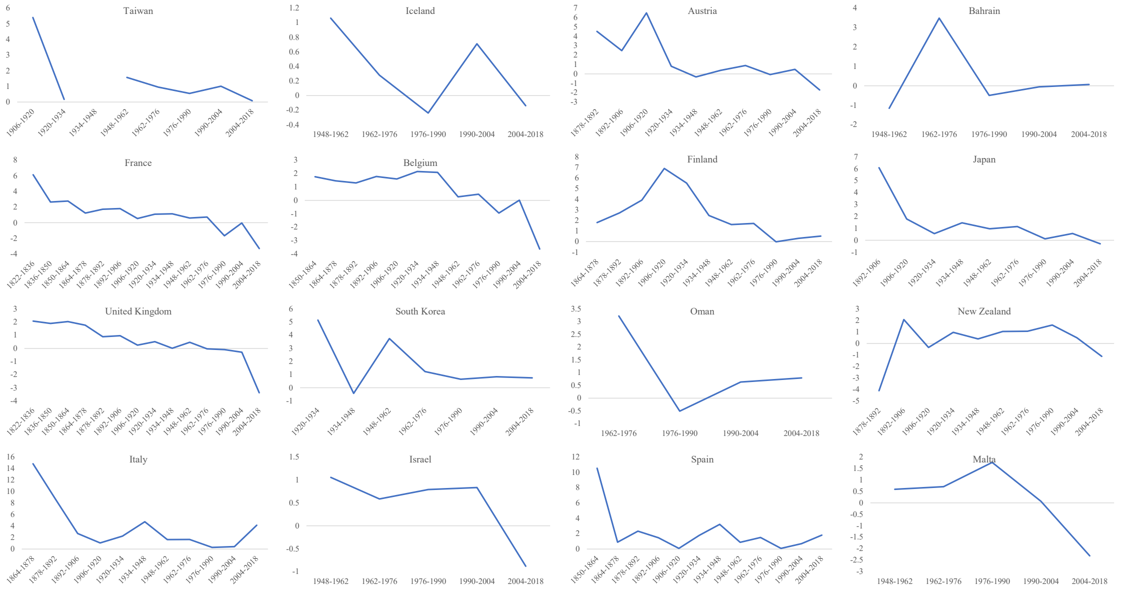

Decile 8:

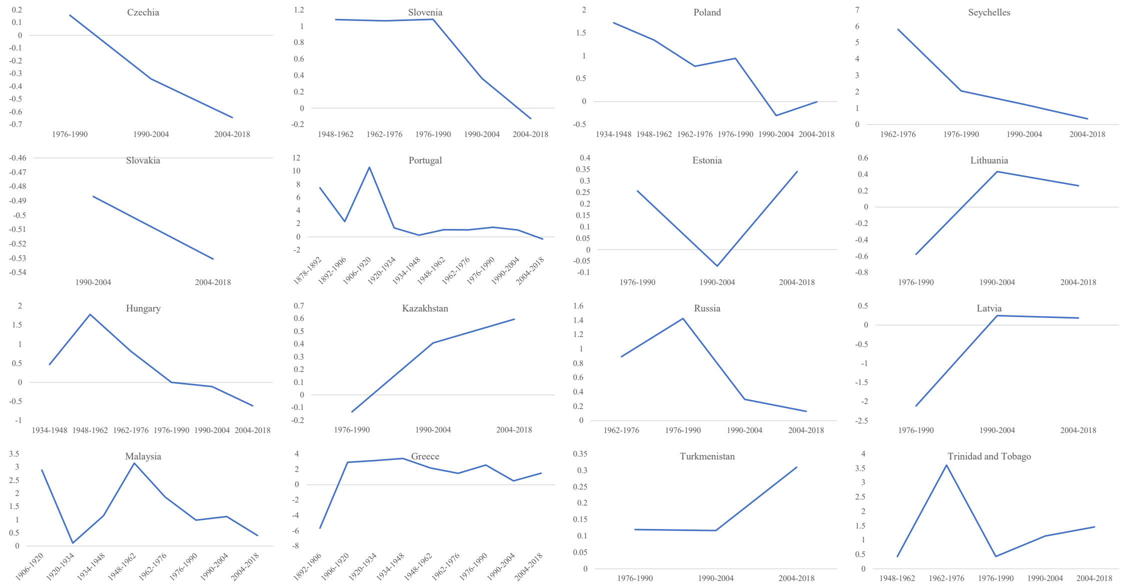

Decile 7:

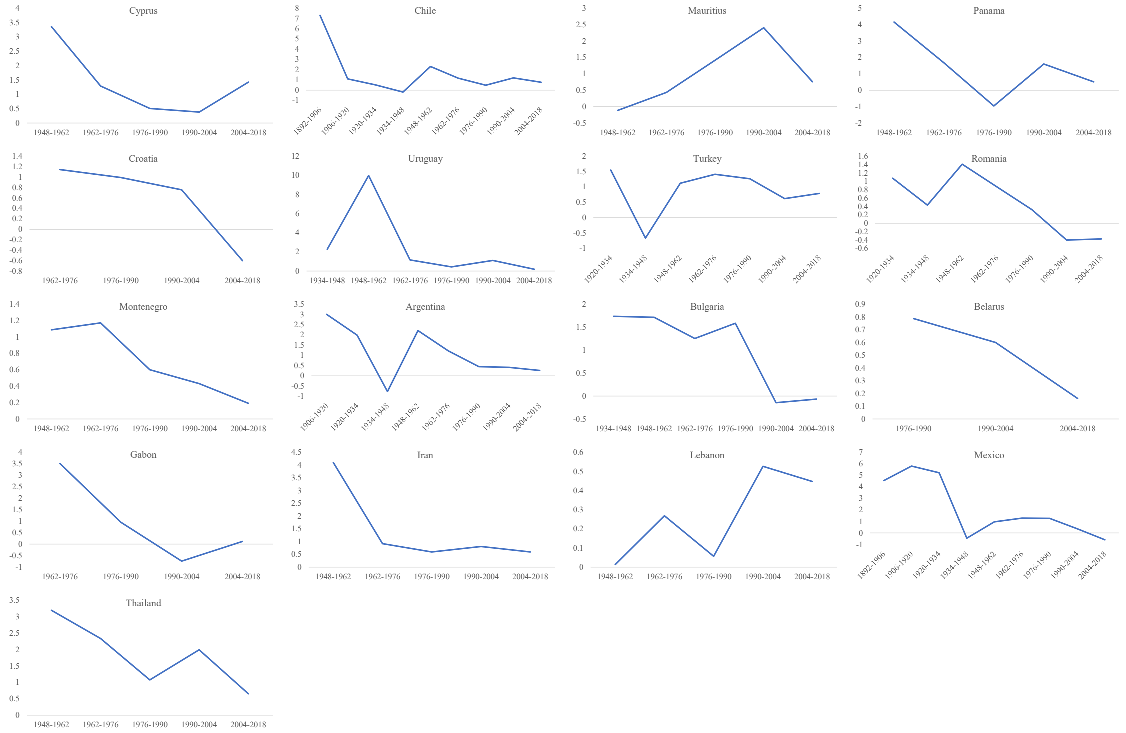

Decile 6:

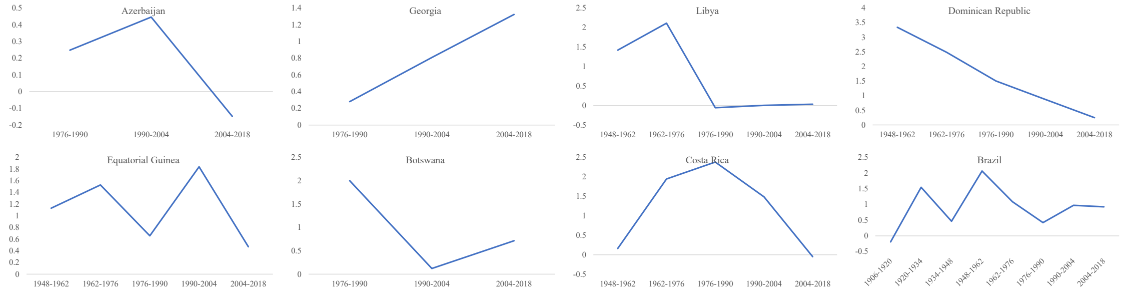

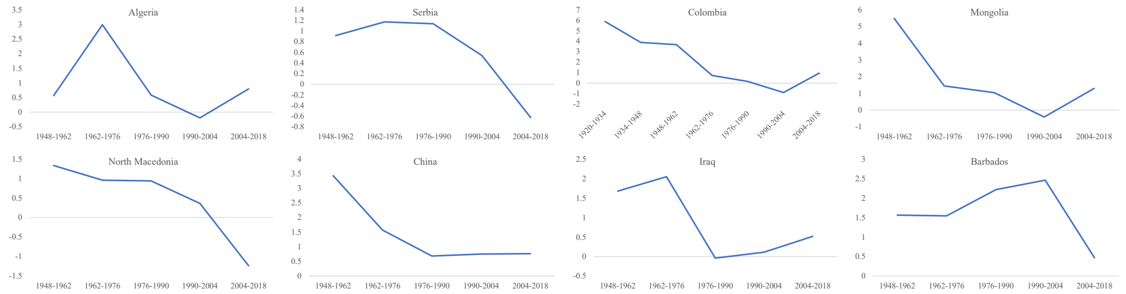

Decile 5:

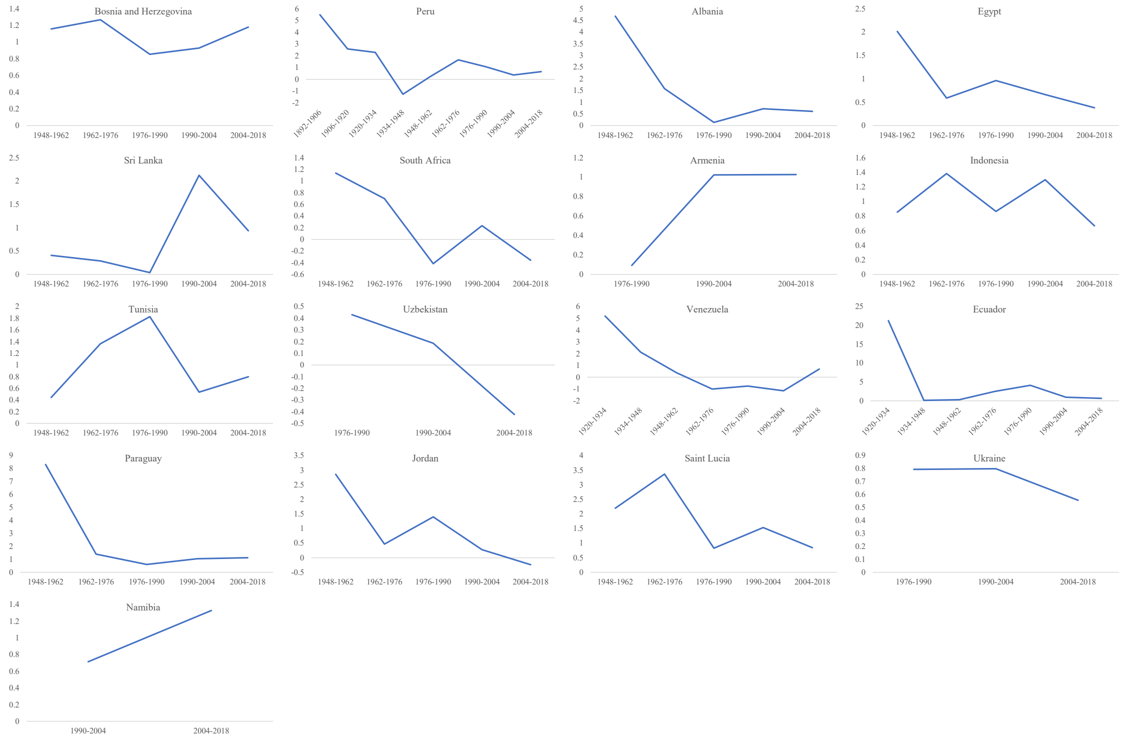

Decile 4:

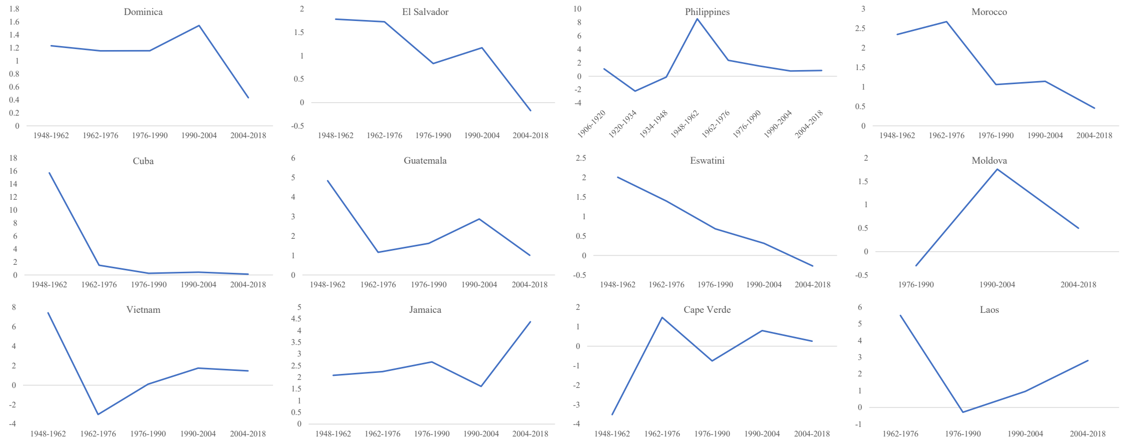

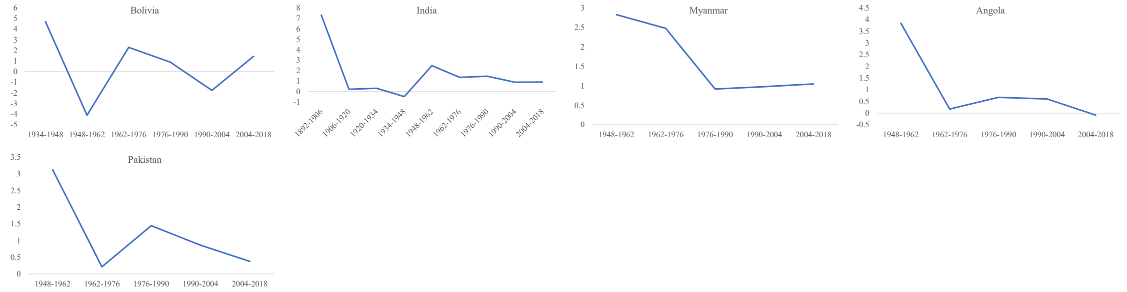

Decile 3:

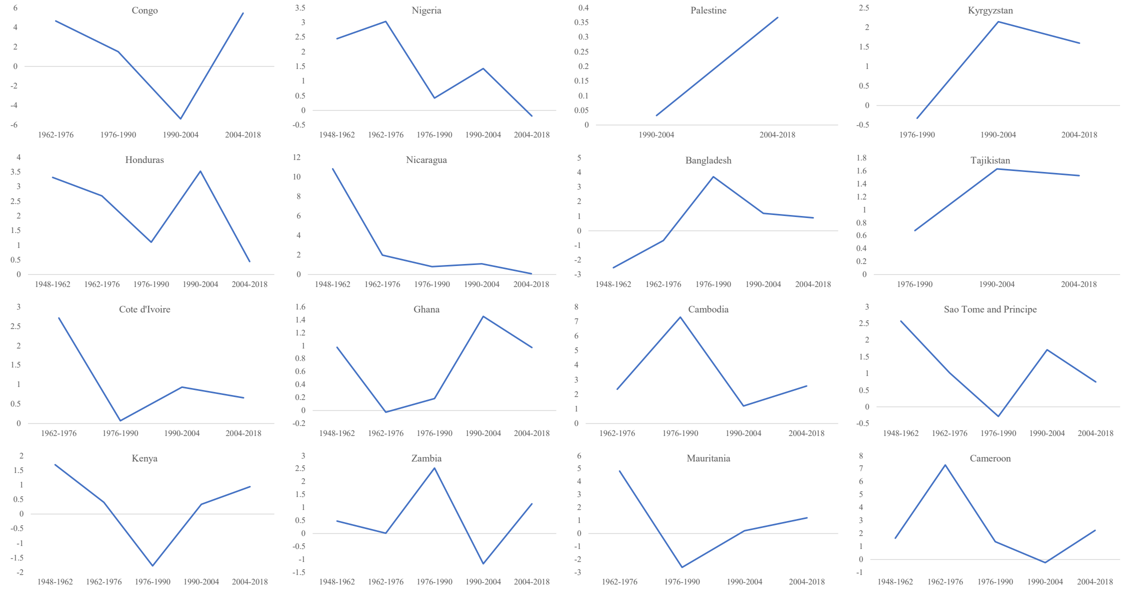

Decile 2:

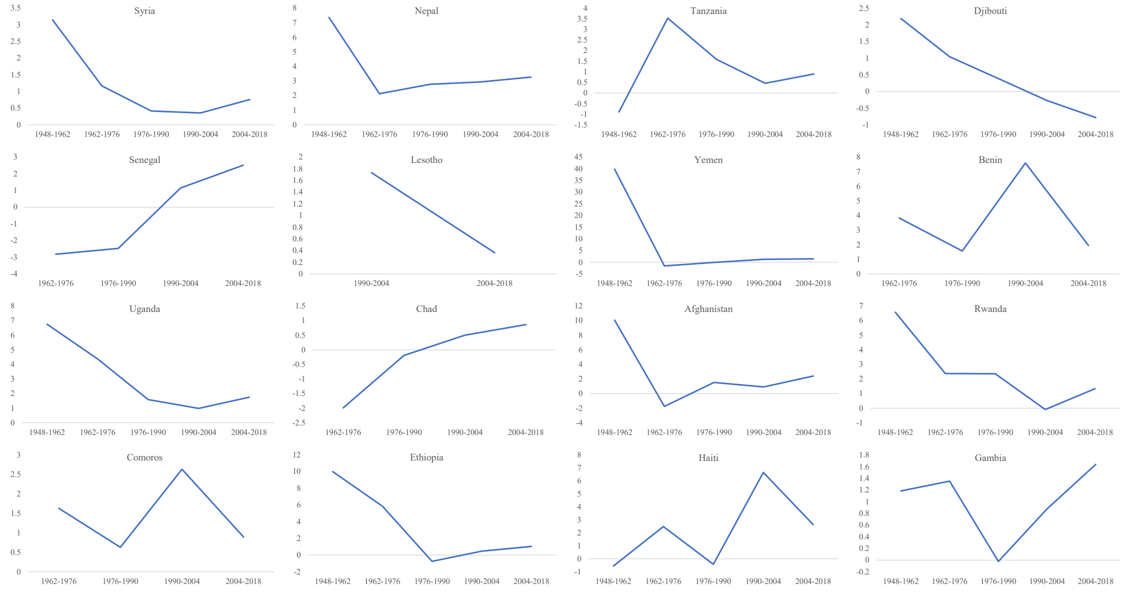

Decile 1:

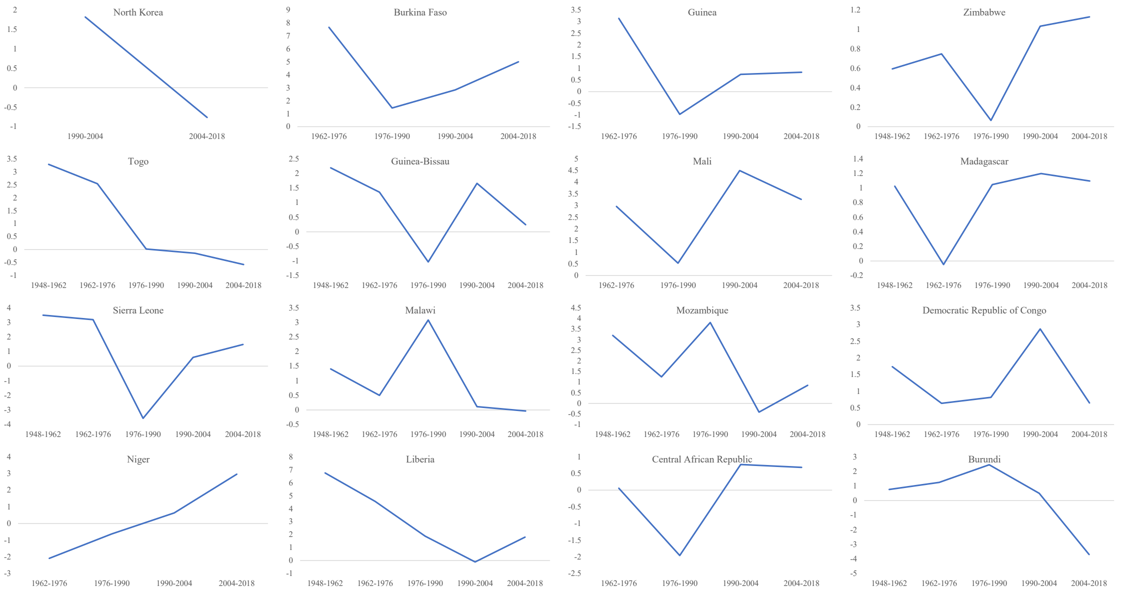
North Korea
1990-2004
2004-2018
Burkina Faso
1962-1976
1976-1990
1990-2004
2004-2018
Guinea
1962-1976
1976-1990
1990-2004
2004-2018
Zimbabwe
1948-1962
1962-1976
1976-1990
1990-2004
2004-2018
Togo
1948-1962
1962-1976
1976-1990
1990-2004
2004-2018
Guinea-Bissau
1948-1962
1962-1976
1976-1990
1990-2004
2004-2018
Mali
1962-1976
1976-1990
1990-2004
2004-2018
Madagascar
1948-1962
1962-1976
1976-1990
1990-2004
2004-2018
Sierra Leone
1948-1962
1962-1976
1976-1990
1990-2004
2004-2018
Malawi
1948-1962
1962-1976
1976-1990
1990-2004
2004-2018
Mozambique
1948-1962
1962-1976
1976-1990
1990-2004
2004-2018
Democratic Republic of Congo
1948-1962
1962-1976
1976-1990
1990-2004
2004-2018
Niger
1962-1976
1976-1990
1990-2004
2004-2018
Liberia
1948-1962
1962-1976
1976-1990
1990-2004
2004-2018
Central African Republic
1962-1976
1976-1990
1990-2004
2004-2018
Burundi
1948-1962
1962-1976
1976-1990
1990-2004
2004-2018

Appendix IV. Results of EKC estimates with additional regressors by country.

Table A3. EKC by country with additional regressors: GDP per capita, trade openness (share of exports and imports in GDP) and urban population (%), for the period 1822–2018. Countries ordered by 2018 GDP per capita.

Country	GDP per capita	Trade openness	Urban population (%)	Intercept
Qatar	-0.0698	1.5401	0.1167	-2.8450
Norway	-0.1797	0.1323	3.1489	-10.0779
United Arab Emirates	0.3792	-0.7644	-2.8582	15.4362
Singapore	0.4989	0.6904	-1.4913	0
Ireland	0.1698	-0.4562	3.1167	-9.9769
Kuwait	0.3114	1.9260	1.3044	-14.8479
Switzerland	0.0993	-0.5844	11.8001	-47.3692
Luxembourg	0.0718	-0.4879	-1.0033	9.5328
United States	0.0292	0.1020	-0.4093	4.1328
Hong Kong	0.9864	-0.5205	0.2242	-6.6668
Australia	0.2470	0.3573	6.7725	-31.1531
Saudi Arabia	0.1232	0.3607	0.8801	-3.9091
Netherlands	1.3344	-0.2932	-2.8583	2.2494
Germany	0.8768	-0.6976	-4.7408	16.6472
Denmark	0.5220	-1.6014	1.8619	-4.4505
Sweden	-1.2464	-0.1536	7.5815	-18.1562
Canada	-0.1915	0.3415	2.2286	-6.3078
Taiwan	0.4970	0.3375	0.6395	-7.1413
Iceland	0.2182	0.0654	1.3220	-6.2772
Austria	0.6686	-0.0376	2.6167	-15.4238
Bahrain	-0.0827	-0.0365	7.8708	-31.1218
France	-1.0045	-0.2945	5.6401	-10.9486
Belgium	1.9619	-0.8805	-29.7554	122.4891
Finland	-0.9210	0.0455	5.1517	-10.9008
Japan	0.8205	-0.1981	0.1067	-6.0650
United Kingdom	-0.0738	-0.0751	-6.8964	33.4421
South Korea	0.3656	0.3964	1.2956	-8.7842
Oman	-0.0086	1.0347	1.8685	-10.0549
New Zealand	0.3282	0.3064	2.1828	-12.2685
Italy	0.4059	-0.9504	9.8154	-39.8952
Israel	0.5495	0.1251	1.1353	-9.1143
Spain	0.1479	-0.2122	4.4872	-18.2761
Malta	0.9647	-0.4053	-11.8359	47.9927
Czechia	0.0294	-0.3829	1.2298	-1.3161
Slovenia	0.5784	-0.0419	-4.5674	14.4784
Poland	0.1297	-0.4413	-1.0241	7.0214
Seychelles	0.6141	-0.3825	5.2838	-23.4955
Slovakia	-0.0095	-0.2699	2.7781	-7.6983
Portugal	1.4416	-0.2794	-0.6216	-9.1165
Estonia	0.5791	-0.2106	11.5359	-50.9418
Lithuania	0.3248	-0.3758	21.6680	-90.9953
Hungary	0.2577	-0.3940	-1.7481	8.4870
Kazakhstan	0.2588	-0.1232	5.7316	-22.4957
Russia	0.0231	-0.1961	-3.8105	19.3672
Latvia	0.3971	-0.0169	34.7299	-149.0457
Malaysia	0.5067	0.1403	1.2864	-9.1343
Greece	1.1920	-0.6569	3.1305	-20.6548
Turkmenistan	0.2526	0.1406	1.1467	-5.1548
Trinidad and Tobago	1.1149	0.4942	1.5470	-16.4798
Cyprus	0.5733	0.8420	0.7405	-10.9222
Chile	0.9094	-0.2507	0.0919	-6.9044
Mauritius	1.4576	0.0594	0.1103	-13.9067
Panama	0.7474	0.5003	-0.2221	-7.8087
Croatia	0.9767	-0.2139	-6.0626	17.2762
Uruguay	0.7183	0.0688	-3.0350	7.2012
Turkey	0.5166	-0.1368	1.3901	-8.9210
Romania	-0.3541	0.2197	0.2144	3.2177
Montenegro	0.3028	0.2062	-0.1280	-2.0974
Argentina	0.5073	-0.1176	1.4812	-9.7674
Bulgaria	0.1816	-0.0713	-1.2653	5.9415
Belarus	0.7752	-0.1491	-5.2888	17.8914
Gabon	-0.3807	2.4956	1.4226	-12.0980
Iran	0.5990	0.1542	0.8308	-7.8577
Lebanon	0.2910	-0.3020	1.5123	-6.8554
Mexico	-0.1237	-0.1021	2.8156	-9.1881
Thailand	2.3443	-0.7810	-0.5320	-15.0889
Azerbaijan	-0.2544	0.4456	5.6360	-20.7098
Georgia	0.0730	0.6676	6.6268	-29.6371
Libya	0.0524	-0.1173	-0.1551	2.8672
Dominican Republic	-0.1154	0.4136	2.4210	-10.1919
Equatorial Guinea	1.7165	-0.0860	-0.6249	-12.3867
Botswana	-0.4194	0.8939	1.8431	-6.8886
Costa Rica	1.1997	0.7480	-0.4079	-12.2515
Brazil	0.7615	-0.0799	0.4221	-8.0240
Algeria	0.3831	-0.3594	1.4064	-6.5830
Serbia	0.0704	0.3719	-5.8901	23.1361
Colombia	0.1328	-0.5376	1.2411	-4.1704
Mongolia	0.4039	-0.6887	2.3567	-8.3743
North Macedonia	-1.2304	0.0160	-6.6157	39.5790
China	0.1457	0.2592	1.2204	-5.2847
Iraq	0.1642	-0.1513	1.5786	-6.1431
Barbados	1.8941	-0.2695	-1.9157	-8.4584
Bosnia and Herzegovina	1.2903	-0.8602	-1.8261	0.6660

Peru	0.8374	-0.1351	0.0725	-6.9950
Albania	2.5299	-0.5467	-5.3809	1.0819
Egypt	0.6989	0.1109	0.9715	-9.6476
Sri Lanka	0.9582	-0.2890	-2.2983	-1.1944
South Africa	0.5191	-0.5173	0.0412	-0.6933
Armenia	1.0734	0.0256	6.4904	-36.3112
Indonesia	0.7698	0.2370	0.4316	-8.9421
Tunisia	0.0409	0.2038	2.2693	-9.9635
Uzbekistan	-0.2779	0.1005	-0.8493	6.7724
Venezuela	-0.4442	0.1142	-0.1565	6.2761
Ecuador	0.8796	-0.5176	2.4120	-14.9862
Paraguay	1.3040	0.2448	0.1996	-13.5867
Jordan	0.2430	0.5568	1.2520	-9.2199
Saint Lucia	1.0501	0.0692	-0.0516	-8.7492
Ukraine	0.5027	-0.4189	-15.5187	64.7551
Namibia	0.1658	-0.0008	1.0351	-5.0464
Dominica	1.2794	-0.1041	-0.3817	-8.7108
El Salvador	0.8097	0.1581	1.0959	-12.2914
Philippines	0.6888	0.1971	0.6852	-9.4094
Morocco	0.0782	0.4252	1.7958	-9.3517
Cuba	0.0823	0.4976	1.4951	-8.0797
Guatemala	0.6658	0.2679	1.2400	-11.9291
Eswatini	0.4574	0.1236	-0.2288	-3.9311
Moldova	1.2741	0.3886	13.3028	-62.3006
Vietnam	-1.5018	2.0827	3.0698	-7.8903
Jamaica	1.7092	-0.2911	0.8855	-15.9835
Cape Verde	0.4373	-0.6132	0.9862	-5.1014
Laos	4.4028	0.3859	-4.9667	-22.0925
Bolivia	0.7704	0.2308	1.7706	-14.4019
India	0.5946	-0.1318	2.1324	-11.4826
Myanmar	0.5852	0.0569	-0.0926	-5.9663
Angola	0.4184	0.1574	-0.0127	-4.2368
Pakistan	0.2936	-0.0707	1.8539	-9.0206
Congo	2.2050	-0.3400	-2.6415	-6.8371
Nigeria	0.0295	-0.8429	2.1658	-5.6060
Palestine	-0.0979	0.2443	6.2840	-27.8600
Kyrgyzstan	0.8181	0.4450	14.8733	-61.6661
Honduras	0.7700	0.3501	0.5842	-10.4051
Nicaragua	0.6642	-0.1639	2.7852	-16.1833
Bangladesh	0.6367	0.1498	0.7220	-9.0825
Tajikistan	0.4976	-0.2944	7.8670	-29.2548
Cote d'Ivoire	1.0517	0.1420	0.5810	-11.9454
Ghana	0.5293	-0.0266	0.7685	-7.9763
Cambodia	1.9622	0.2965	1.0829	-21.0829
Sao Tome and Principe	1.0840	0.4050	0.4140	-12.7396
Kenya	0.3602	0.0020	-0.1946	-3.4496
Zambia	-0.0134	1.9322	-0.1513	-8.9206
Mauritania	-0.1756	0.2857	0.9019	-3.7706
Cameroon	1.3118	0.8874	1.0774	-18.9682
Syria	0.7907	-0.1276	4.0307	-21.4022
Nepal	1.3247	0.1666	0.8807	-14.9247
Tanzania	1.0551	-0.2712	-0.3271	-7.7597
Djibouti	0.8881	-0.3667	2.1779	-15.0247
Senegal	0.3094	0.3544	1.1359	-8.7301
Lesotho	-0.1536	-0.1518	0.6151	0.0701
Yemen	1.7403	-0.3175	0.3244	-14.4699
Benin	3.9717	-0.1361	0.1834	-31.2815
Uganda	1.7984	-0.1640	-0.1879	-14.4673
Chad	0.7584	-0.0468	0.9398	-10.6246
Afghanistan	0.8529	-0.7121	1.1081	-8.5041
Rwanda	1.2685	0.4897	0.3596	-14.3893
Comoros	0.9453	-0.2811	0.8218	-10.1237
Ethiopia	0.9109	0.0595	1.4367	-13.0156
Haiti	2.4417	0.1204	1.0416	-23.9774
Gambia	0.4584	0.2683	0.8368	-9.2552
North Korea	0.2658	0.2964	-25.4156	102.1287
Burkina Faso	0.2516	-0.0765	1.2685	-7.5366
Guinea	0.0003	0.3035	0.1201	-3.2347
Zimbabwe	0.9185	-0.0591	-0.5213	-4.8348
Togo	-0.1440	0.4469	1.2574	-6.6426
Guinea-Bissau	1.1570	0.4550	0.1915	-12.5295
Mali	0.4024	0.6637	1.0397	-11.3497
Madagascar	1.7492	-0.3154	0.9418	-16.6464
Sierra Leone	1.2776	0.1642	-0.3509	-10.6820
Malawi	0.5721	-0.0429	-0.5525	-4.9140
Mozambique	0.2213	0.9583	-1.6945	-1.9877
Democratic Republic of Congo	1.2736	-0.1243	-1.6109	-5.2632
Niger	1.7816	0.1868	2.5888	-22.2620
Liberia	0.5244	0.3222	-0.5520	-4.4414
Central African Republic	1.2496	0.3039	1.0243	-16.4140
Burundi	1.2398	-0.4729	0.6943	-11.4337

Note: data obtained from ourworldindata.org.
